# Faithful modeling of terminal CD8^+^T cell dysfunction and epigenetic stabilization in vitro

**DOI:** 10.1172/jci.insight.191220

**Published:** 2025-10-08

**Authors:** Amir Yousif, Abbey A. Saadey, Ava Lowin, Asmaa M. Yousif, Ankita Saini, Madeline R. Allison, Kelley Ptak, Eugene M. Oltz, Hazem E. Ghoneim

**Affiliations:** 1 Department of Microbial Infection and Immunity, College of Medicine;; 2 Molecular, Cellular, and Developmental Biology Graduate Program;; 3 Biomedical Sciences Graduate Program; and; 4 The Pelotonia Institute for Immuno-Oncology, James Comprehensive Cancer Center, The Ohio State University, Columbus, Ohio, USA.

**Keywords:** Immunology, Oncology, Adaptive immunity, Cancer immunotherapy, Epigenetics

## Abstract

Epigenetic scarring of terminally dysfunctional (T_Dysf_) CD8^+^ T cells hinders long-term protection and response to immune checkpoint blockade during chronic infections and cancer. We developed a faithful in vitro model for CD8^+^ T cell terminal dysfunction as a platform to advance T cell immunotherapy. Using TCR-transgenic CD8^+^ T cells, we found that 1-week peptide stimulation, mimicking conditions in previous models, failed to induce a stable exhaustion program. In contrast, prolonged stimulation for 2–3 weeks induced T cell dysfunction but triggered activation-induced cell death, precluding long-term investigation of exhaustion programs. To better mimic in vivo exhaustion, we provided post-effector, chronic TGF-β1 signals, enabling survival of chronically stimulated CD8^+^ T cells for over 3 weeks. These conditions induced a state of terminal dysfunction, marked by a stable loss of effector, cytotoxicity, and memory programs, along with mitochondrial stress and impaired protein translation. Importantly, transcriptomic and epigenetic analyses verified the development of terminal exhaustion-specific signatures in T_Dysf_ cells. Adoptive transfer of T_Dysf_ cells revealed their inability to recall effector functions or proliferate after acute lymphocytic choriomeningitis virus rechallenge. This tractable model system enables investigation of molecular pathways driving T cell terminal dysfunction and discovery of therapeutic targets for cancer or chronic infections.

## Abstract 2

Here, we developed the first faithful *in vitro* model of terminal CD8 T cell dysfunction that enables investigation of epigenetic scarring of exhausted T cells

## Graphical abstract

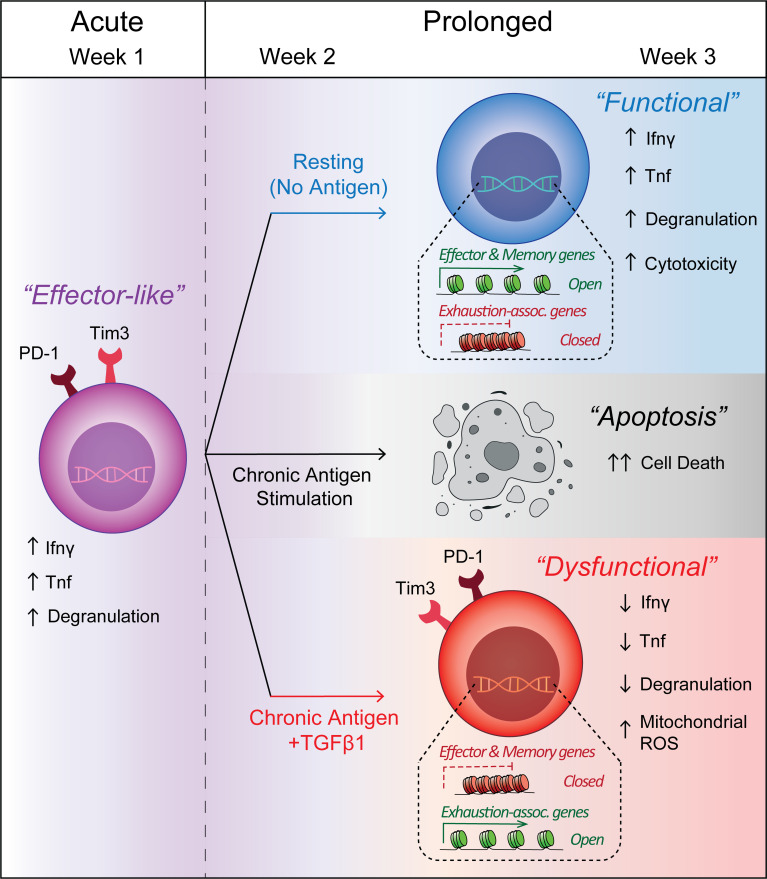

## Introduction

CD8^^+^^ T cell effector function is critical for clearing viral infections or tumor cells. When antigen-primed CD8^^+^^ T cells experience prolonged antigenic stimulation and inflammatory microenvironments, they progressively lose their cytotoxic capacity and become dysfunctional. This state of T cell dysfunction, also called exhaustion, is characterized by high expression of surface inhibitory receptors, as well as a gradual loss of effector functions, proliferation capacity, and memory potential, along with impaired mitochondrial function and protein translation (1, 2). The gradual progression of dysfunction, coupled with variations in spatial cues, produces heterogeneous exhausted T cell (TEX) populations (2–5). These include a progenitor subset with self-renewal and effector capacity; a cytolytic subset with strong cytotoxic function; and a terminally exhausted subset with poor function, survival, and proliferative capacities (1–3, 6). Immune checkpoint blockade (ICB) is a revolutionary cancer immunotherapy that reinvigorates both the progenitor and cytolytic TEX subsets by blocking signals from inhibitory receptors, such as cytotoxic T lymphocyte antigen 4 and programmed cell death 1 (PD-1), but has limited impact on terminally exhausted T cells — a major obstacle for ICB therapy in many patients with cancer.

We and others have shown that the epigenome plays a central role in locking TEX fate commitment (3, 7–14). Importantly, de novo DNA methylation enforces the silencing of effector and memory programs in TEX cells, restraining ICB response (8). Recent studies have revealed key roles for microenvironmental signals in regulating T cell dysfunction, such as strength and duration of TCR stimulation, chronic TGF-β1 exposure, type I IFN signaling, and nutrient limitation (1, 2). Additionally, hypoxia — adapted for in vitro modeling (15) — showed a key role in driving and potentially accelerating T cell dysfunction (16, 17). Yet the specific signals critical for initiating and imprinting exhaustion-associated epigenetic programs remain unclear. This major gap in the field is due to the diverse range of cues that CD8^^+^^ T cells encounter in vivo within tumor or infected tissue microenvironments. This complexity makes it challenging to isolate and understand the individual and combined effects of exhaustion-inducing signals on epigenetic regulation of TEX cells.

As such, reductionist approaches will be critical for dissecting how signals in each microenvironment drive or counteract epigenetic programming of T cell exhaustion. Recent efforts to develop in vitro models of T cell exhaustion have aimed to address these challenges (18–21). In previous models, CD8^^+^^ T cells acquire exhaustion-like features, including inhibitory receptor upregulation, reduced proliferation, and mitochondrial stress. However, several models rely primarily on persistent TCR stimulation as the sole driver of T cell exhaustion, with repeated antigen-dependent or independent (anti-CD3) stimulation under a short timeline of 5–10 days (18–21). The fundamental obstacle we have found in TCR-based approaches is that repeated TCR stimulations of CD8^^+^^ T cells leads to activation-induced cell death (9, 20). Thus, persistent TCR signaling alone fails to replicate the extended timeline of T cell exhaustion observed during chronic infection or cancer, in which at least 2–3 weeks of chronic stimulation in the presence of other microenvironmental signals is necessary to establish a terminally dysfunctional (T__Dysf__) state (11, 22–24). Furthermore, it remains unclear whether in vitro–induced dysfunction is stable after antigen withdrawal — a core defining feature of the epigenetically fixed state of terminal exhaustion observed in vivo (11, 22–24). Therefore, it is essential to develop a robust model system that faithfully recapitulates the progressive nature of T cell exhaustion and enables tracking of chronically stimulated T cells over extended periods. Such a system is vital for developing reliable discovery platforms to identify novel therapeutic targets aimed at preventing or reversing T cell dysfunction and enhancing the effectiveness of T cell immunotherapies.

Here, we developed an antigen-dependent model system of T cell exhaustion using TCR-transgenic, lymphocytic choriomeningitis virus–glycoprotein 33-41–specific (LCMV-GP33–specific) CD8^^+^^ T cells. We found that prolonged (2–3 weeks) rather than short-term antigen stimulation (1 week) was required to induce stable T cell exhaustion. To overcome activation-induced cell death and better mimic in vivo conditions, we introduced chronic TGF-β1 signals at the post-effector phase, which supported CD8^^+^^ T cell survival. The combined signals and long-term viability faithfully induced terminal dysfunction in CD8^^+^^ T cells (T__Dysf__), which displayed a remarkable loss of effector and memory programs, mitochondrial stress, and impaired protein translation. Transcriptome and epigenome analyses of these dysfunctional lymphocytes verified the acquisition of exhaustion-specific signatures. Furthermore, adoptive T cell transfer studies showed that T__Dysf__ cells had impaired effector recall responses and proliferation capacity. Our model enables investigation of the long-term developmental process that leads to terminal dysfunction in antigen-specific CD8^^+^^ T cells, providing a faithful platform for identifying new therapeutic targets to block or reverse epigenetic processes stabilizing terminal dysfunction.

## Results

### *Acute antigenic stimulation fails to induce a dysfunctional program in CD8^^+^^ T cells*.

**Figure 1:**
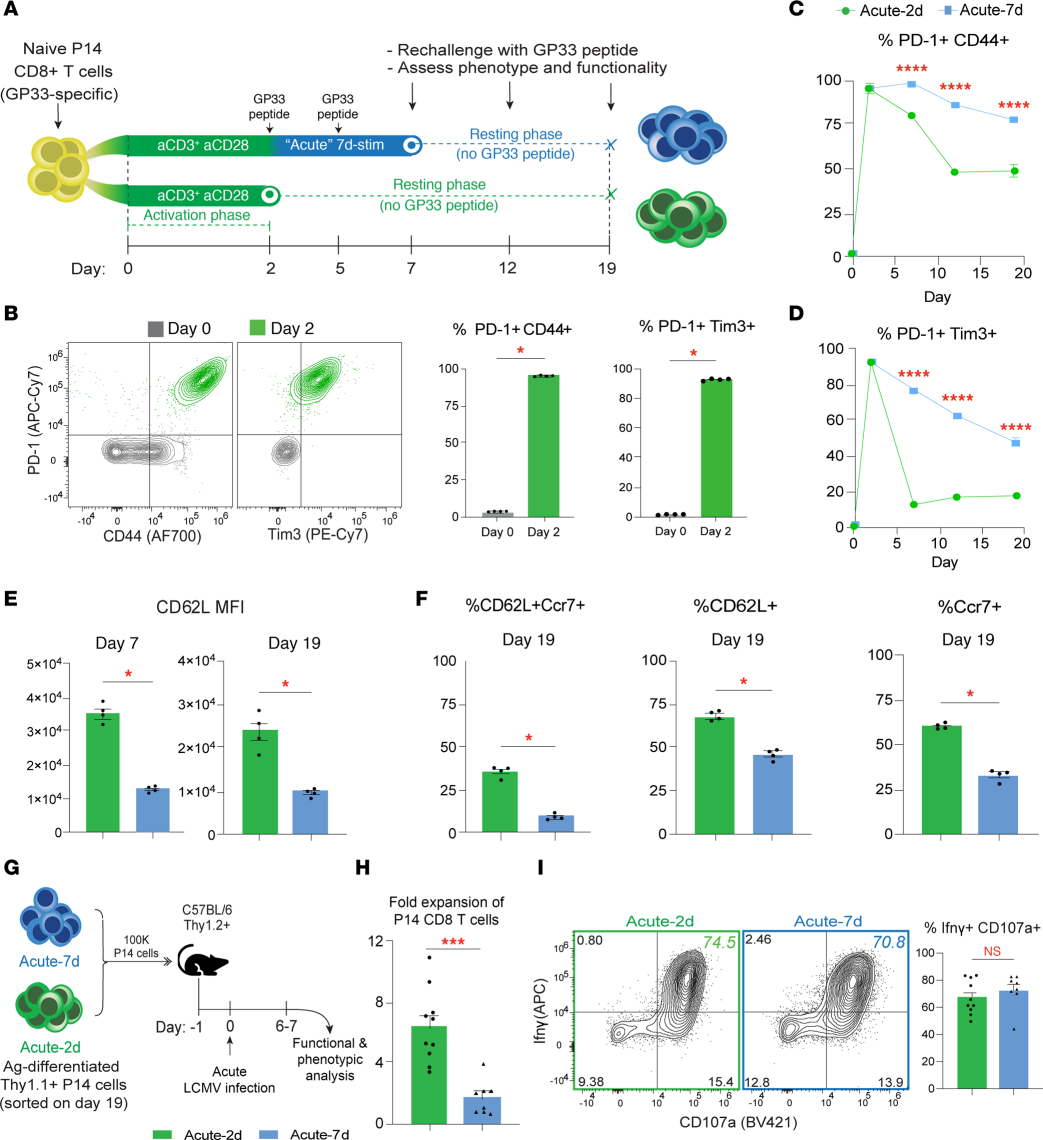
Acute antigenic stimulation for 1 week fails to induce a dysfunctional program in CD8^+^ T cells. (**A**) Schematic for in vitro stimulation of GP33-specific P14 CD8^+^ T cells isolated from spleens of naive mice. P14 cells were activated with anti-CD3 and anti-CD28 day 0–2, followed by resting (“Acute-2d”), or by 2 rounds of GP33 peptide stimulation day 2–7 followed by resting until day 19 (“Acute-7d”). (**B**) Representative FACS plots showing expression of PD-1 and CD44 or Tim3 on P14 cells from day 0 to day 2 and summary bar graphs showing frequency of PD-1^+^CD44^+^ or PD-1^+^Tim3^+^ P14 cells on days 0 and 2. (**C**) Longitudinal tracking of % PD-1^+^CD44^+^ P14 cells for either Acute-2d (bottom line, green) or Acute-7d (top line, blue) or (**D**) % PD-1^+^Tim3^+^ P14 cells from day 0–19. (**E**) Expression levels (shown by geometric mean fluorescence intensity, gMFI) of CD62L from Acute-2d or Acute-7d P14 cells on days 7 and 19. (**F**) Bar graphs showing % of CD62L^+^Ccr7^+^, total CD62L^+^, and total Ccr7^+^ P14 cells on day 19. (**G**) Schematic for adoptive transfer of 100K Acute-2d or Acute-7d in vitro–stimulated P14 cells in congenically distinct C57BL/6 mice on day 19, followed by acute LCMV infection and analysis day 6–7 postinfection. (**H**) Fold expansion of P14 cells relative to endogenous GP33-specific CD8^+^ T cells on day 6–7 postinfection. (**I**) Representative FACS plots and % Ifnγ^+^CD107a^+^ P14 cells after ex vivo GP33 peptide restimulation of splenocytes. For **B**–**F** *n* = 4 biological replicates, representative of 2 to 3 independent experiments. For **H** and **I**, data were pooled from 2 independent experiments, each with *n* = 3–4 biological replicates per group. *P < 0.05, ***P < 0.001, ****P < 0.0001. Comparisons were determined by 2-way ANOVA (**C** and **D**) or Mann–Whitney *U* test (unpaired, 2-sided) (**B**, **E**, **F**, **H**, and **I**). Error bars indicate mean ± SEM.

To determine whether 1 week of repeated TCR stimulation induces a stable dysfunctional program in CD8^^+^^ T cells, we utilized naive TCR-transgenic murine CD8^^+^^ T cells, “P14 cells,” which respond to LCMV-derived GP33 peptide. We compared P14 cells initially activated using anti-CD3 plus anti-CD28 stimulation on day 0–2 (Acute-2d) versus those that received repeated GP33 peptide stimulations following this activation phase until day 7 (Acute-7d) (Figure 1A). After initial activation (days 0–2), over 98% of P14 cells upregulated CD44 and PD-1, with most coexpressing PD-1 and Tim3 — indicating robust effector differentiation (Figure 1, B–D, and Supplemental Figure 1, A and B; supplemental material available online with this article; https://doi.org/10.1172/jci.insight.191220DS1). By day 7, repeated stimulation maintained high frequencies of CD44^^+^^PD-1^^+^^ and PD-1^^+^^Tim3^^+^^ populations in Acute-7d cells (Figure 1, C and D, and Supplemental Figure 1B), mimicking inhibitory receptor upregulation on effector CD8^^+^^ T cells in vivo (25). To assess stability of these features, we rested antigen-experienced P14 cells for 12 days without GP33 stimulation and rechallenged them with GP33 peptide on days 12 and 19 (Figure 1A). The frequencies of CD44^^+^^PD-1^^+^^ and PD-1^^+^^Tim3^^+^^ populations gradually declined over time but remained elevated in Acute-7d P14 cells versus Acute-2d cells by day 19 (Figure 1, C and D, and Supplemental Figure 1, B–D). These findings suggest that Acute-7d P14 cells do not have stable maintenance of inhibitory receptors following rest, unlike TEX cells retaining high levels of inhibitory receptors in vivo (26, 27).

A hallmark of T cell exhaustion is the failure to restore memory programs after antigen removal (11–14, 24). To determine whether repeated antigen stimulation for 1 week affects the recovery of memory programs, we tracked expression levels of memory- and stemness-associated programs before and after resting (Ly108, L selectin [CD62L], Ccr7, and IL-7 receptor [Il7r; CD127]). On day 7, Acute-7d P14 cells initially downregulated Ly108 and CD62L, but Ly108 was recovered after resting while CD62L remained higher in Acute-2d cells (Figure 1E and Supplemental Figure 1I). The frequencies of CD62L^^+^^ Ccr7^^+^^ cells were higher in rested Acute-2d cells versus Acute-7d cells, while Il7r remained high in both groups by day 19 (Figure 1F and Supplemental Figure 1J). Notably, the proliferative activity (measured by Ki67 expression) was comparable between the two groups on day 19, with a modest but nonsignificant increase in Acute-2d cells (Supplemental Figure 1H). These findings suggest that 1 week of repeated stimulation does not lead to a loss of memory features, mirroring the retained memory potential in effector CD8^^+^^ T cells isolated 1 week following chronic LCMV infection (24).

We next evaluated effector function after GP33 rechallenge at different time points. Both groups exhibited high polyfunctionality (Ifnγ and Tnf coexpression, CD107a: degranulation activity), which increased with resting (Supplemental Figure 1, G and K–N). Acute-7d P14 cells had significantly higher degranulation activity and increased expression of cytotoxic molecules granzyme B (Gzmb) and Perforin compared with Acute-2d cells (Supplemental Figure 1, E–G), indicating that repeated acute antigen stimulation may enhance cytolytic effector differentiation in CD8^^+^^ T cells. We also observed lower apoptosis (annexin V) in Acute-2d, while both groups retained viable populations after resting (Supplemental Figure 1O). Additionally, mitochondrial mass (MitoTracker-Green) and oxidative phosphorylation activity (MitoSox) were similar (Supplemental Figure 1, P and Q). Overall, our findings demonstrate that 1 week of antigen stimulation alone is insufficient to induce stable dysfunction, suggesting that longer antigenic stimulation or additional microenvironmental cues are required for terminal exhaustion.

Finally, we tested recall responses by adoptively transferring Acute-2d or Acute-7d CD8^^+^^ T cells (day 19) into mice, followed by acute LCMV infection (Figure 1G), as terminally TEX cells have limited capacity to mount a response to antigen rechallenge (11, 24). Both groups underwent extensive antigen-dependent proliferation, though Acute-2d cells exhibited greater expansion (Figure 1H), possibly due to enhanced survival or CD62L-mediated lymphoid homing (Figure 1, E and F). Yet, after expansion, CD62L expression was comparable between the groups (Supplemental Figure 2B). Upon ex vivo GP33 peptide stimulation, both groups showed strong polyfunctionality, expressing high levels of Ifnγ, CD107a, Perforin, Gzmb, and Tnf (Figure 1I and Supplemental Figure 2, D, E, and H). Markers of proliferation, activation, and memory (CD62L, Ki67, CD44, Ly108, Tcf1, PD-1, Tim3) remained comparably elevated (Supplemental Figure 2, A–C, F, G, I, and J), with similar expression patterns in liver and lungs (Supplemental Figure 2, K–T). Overall, these findings confirm that repeated antigen stimulation for 1 week fails to induce terminal dysfunction, as these cells retain robust recall capacity.

### *Chronic antigenic stimulation faithfully induces terminal dysfunction*.

**Figure 2:**
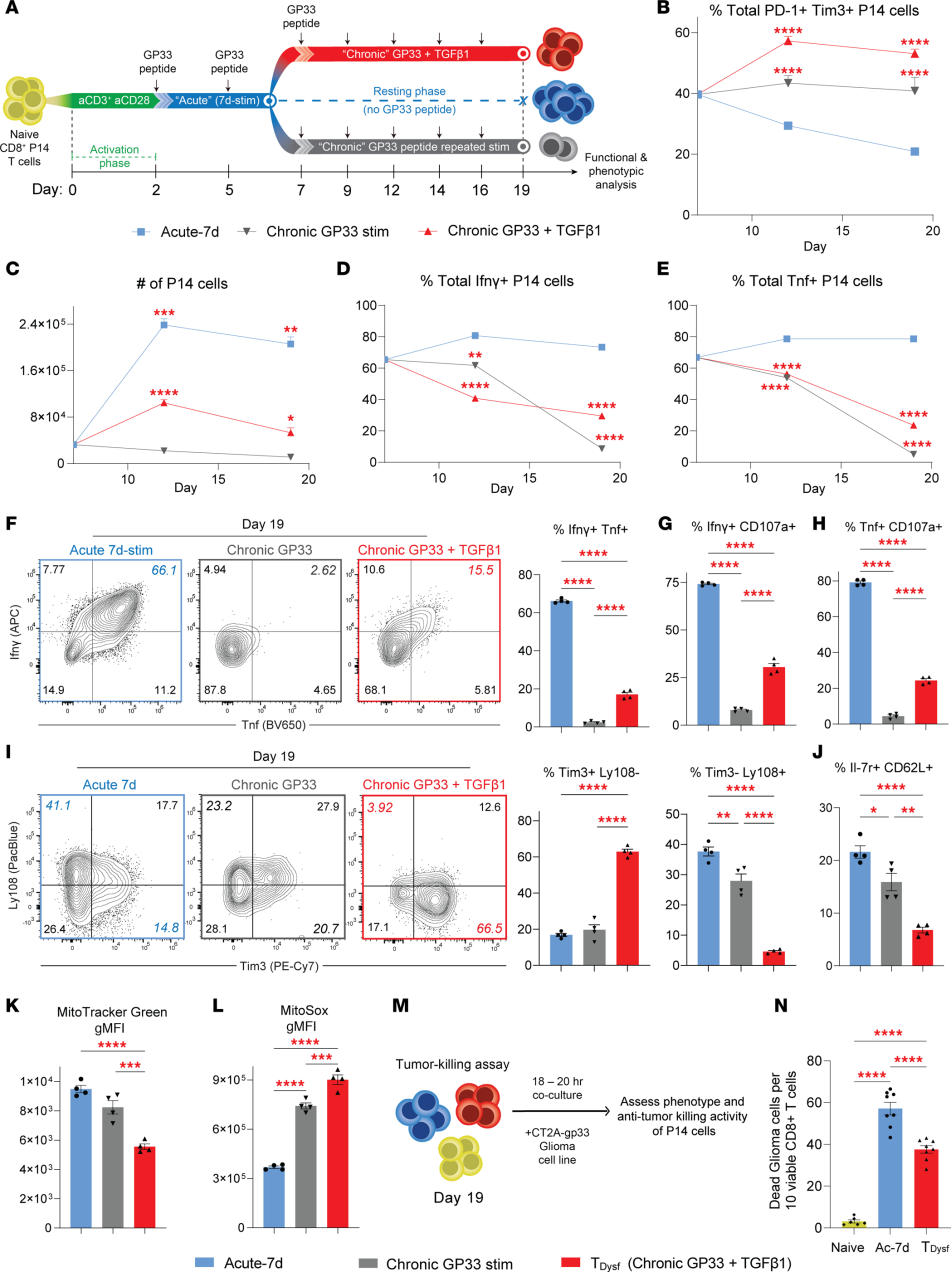
Chronic antigenic stimulation faithfully induces terminal dysfunction. (**A**) Schematic for in vitro stimulation of P14 cells: Activated P14 cells were acutely stimulated by GP33 peptide from day 2–7 and rested until day 19 (Acute-7d condition, blue) or repeatedly GP33-stimulated from day 2–19 with chronic TGF-β1 exposure (day 7–19, red T_Dysf_) or without TGF-β1 signals (gray). Longitudinal tracking of (**B**) frequencies of PD-1^+^Tim3^+^ P14 cells, (**C**) P14 cell numbers/200 μL, (**D**) %Ifnγ^+^ and (**E**) %Tnf^+^ from day 7–19 for Acute-7d (blue), Chronic GP33 stim (gray), or Chronic GP33+TGF-β1 (red). (**F**) Representative FACS plots and bar graphs of Ifnγ and Tnf expression, (**G**) %Ifnγ^+^CD107a^+^, or (**H**) %Tnf^+^CD107a^+^ P14 cells on day 19 after GP33 peptide rechallenge. (**I**) Representative FACS plots and bar graphs of Tim3 and Ly108 expression. (**J**) Summary bar graph of %Il7r^+^CD62L^+^ P14 cells on day 19. Expression level (geometric MFI; gMFI) of (**K**) MitoTracker Green dye (for mitochondrial mass) and (**L**) MitoSox Red dye (for mitochondrial ROS) within P14 cells on day 19. (**M**) Schematic for tumor-killing assay of naive (yellow), Acute-7d, or T_Dysf_ P14 cells cocultured with GP33-expressing CT2A glioma tumor cells. (**N**) Bar graph showing numbers of dead CT2A-GP33 cells per 10 viable P14 cells. All *n* = 4 biological replicates, representative of 2 to 3 independent experiments. Adjusted *P* value *P < 0.05, **P < 0.01, ***P < 0.001, ****P < 0.0001. Comparisons were determined by 2-way ANOVA (**B**–**E**) or 1-way ANOVA with Tukey’s multiple comparisons (**F**–**L** and **N**). Error bars indicate mean ± SEM.

To develop a model system for terminal dysfunction in CD8^^+^^ T cells, we extended the duration of repeated GP33 peptide stimulation for an additional 2 weeks (Chronic GP33 stim) (Figure 2A). Compared with acutely stimulated Acute-7d condition, 2–3 weeks of prolonged antigen stimulation produced a higher frequency of PD-1^^+^^Tim3^^+^^ P14 cells (Figure 2B). However, the chronically stimulated P14 cells exhibited substantial loss of viability, with a progressive decline in total cell numbers and increased cell death, likely due to activation-induced cell death — a major limitation for studying CD8^^+^^ T cells under prolonged periods of TCR stimulation in vitro (20) (Figure 2C and Supplemental Figure 3F).

Previous studies to model T cell exhaustion in vitro have used plate-bound anti-CD3/CD28 stimulation (day 0–2) followed by repeated plate-bound anti-CD3 stimulations for 5–10 days (20, 21). While extended strong TCR stimulation induced high levels of PD-1 and Tim3 (Supplemental Figure 3, A–C), this method also induced extensive cell death that limited reliable functional assessment and tracking of the epigenetic remodeling toward terminal T cell exhaustion (Supplemental Figure 3, A, D, and E). To better mimic in vivo conditions where CD8^^+^^ T cells can survive and develop exhaustion following prolonged antigen exposure, we provided continuous TGF-β1 signals between days 7 and 19 (Chronic GP33 stim + TGF-β1) to ameliorate activation-induced cell death (Figure 2A) (9). Our previous work demonstrated that prolonged, post-effector TGF-β1 exposure drives terminal dysfunction in human CD8^^+^^ T cells that are chronically stimulated in an antigen-independent manner (9). Indeed, we found that chronic GP33 plus TGF-β1–stimulated P14 cells not only showed improved cell viability compared with chronic GP33 alone or plate-bound stimulation approaches (Figure 2C and Supplemental Figure 3, D–F) but also further increased PD-1 and Tim3 coexpression (Figure 2B). To evaluate the impact of TGF-β1 exposure on CD8^^+^^ T cell survival, we performed annexin V and live/dead staining on chronically stimulated CD8^^+^^ T cells. TGF-β1 supplementation significantly improved the viability of P14 T cells under chronic stimulation, with reduced apoptosis and increased proportions of live cells compared with chronic GP33 stimulation alone on both days 14 and 20 (Supplemental Figure 3, F–H). These findings suggest that TGF-β1 attenuates activation-induced cell death. The improved viability of P14 cells under chronic GP33 plus TGF-β1 stimulation (hereafter referred to as T__Dysf__) enabled robust analysis of exhaustion programming during prolonged antigenic stimulation.

We next examined whether chronic GP33 plus TGF-β1 stimulation induces dysfunction characteristic of terminally exhausted CD8^^+^^ T cells. By tracking effector cytokine production in chronically versus acutely stimulated P14 cells, we observed that chronic GP33 stimulation — with or without TGF-β1 — led to a progressive decline in effector cytokine output (Figure 2, D and E), indicative of a transition toward dysfunction. Importantly, T__Dysf__ exhibited a significant loss of polyfunctionality compared with acutely stimulated cells, with reduced frequencies of Ifnγ^^+^^Tnf^^+^^, Ifnγ^^+^^CD107a^^+^^, and Tnf^^+^^CD107a^^+^^ cells, reflecting severe impairment in both cytokine production and degranulation capacity (Figure 2, F–H). Furthermore, T__Dysf__ cells showed substantial loss of memory-associated markers, including reduced expression and frequencies of Il7r and CD62L, compared with both acutely and chronically stimulated controls (Figure 2J and Supplemental Figure 4E), further indicating impaired memory differentiation.

We also examined exhaustion-associated surface markers and found that T__Dysf__ cells exhibited a shift toward a terminally exhausted phenotype. Alongside sustained high coexpression of PD-1 and Tim3, T__Dysf__ cells showed decreased frequencies and expression of Ly108 — a marker of the progenitor subset of TEX cells — leading to an increased proportion of Tim3^^+^^Ly108^^–^^ cells, a phenotype of terminal exhaustion (2, 28, 29) (Figure 2I and Supplemental Figure 4F). Furthermore, T__Dysf__ cells demonstrated increased frequencies of Tim3^^+^^CD62L^^–^^ cells, further supporting their progression toward a terminally exhausted state (Supplemental Figure 4A). Notably, T__Dysf__ cells showed elevated expression of CD103, a well-established downstream target gene of TGF-β1 signaling that marks a terminally exhausted subset of human tumor-reactive CD8^^+^^ T cells (30–33) (Supplemental Figure 4D). In addition, T__Dysf__ cells showed increased expression of Gzmb — commonly associated with terminal dysfunction (28, 34) — as well as higher levels of perforin compared with Acute-7d cells, likely reflecting the effects of sustained TCR stimulation (Supplemental Figure 4, B and C). To assess mitochondrial stress, a key feature linked to terminal dysfunction in CD8^^+^^ T cell exhaustion (15, 21, 35), we tracked changes in mitochondrial functions within T__Dysf__ P14 cells and found a significant reduction in mitochondrial mass accompanied by increased mitochondrial ROS levels — a marker of progressive T cell exhaustion in vivo (Figure 2, K and L). These results indicate that chronic antigen plus TGF-β1 stimulation over 3 weeks establishes a dysfunctional program in CD8^^+^^ T cells.

To assess the cytotoxic function of in vitro–differentiated P14 cells, we utilized an antigen-dependent tumor-killing assay, in which we cocultured naive or chronically or acutely stimulated P14 cells with GP33-expressing CT2A glioma cells for 18–20 hours (Figure 2M). Consistent with their impaired effector functions, T__Dysf__ cells demonstrated significantly reduced tumor-killing activity compared with acutely stimulated P14 cells (Figure 2N), further demonstrating their exhausted state. Overall, these findings reveal a fundamental role for chronic TGF-β1 signals in driving both human and mouse T cell dysfunction (9). Our model system should now enable in vitro studies of how chronically stimulated CD8^^+^^ T cells progress toward dysfunction under extended antigenic stimulation and assessment of microenvironmental cues beyond TCR signaling alone in this process.

### *Chronic TCR plus TGF-β1 signaling establishes the transcriptomic signature of terminal exhaustion and impairs protein translation*.

**Figure 3:**
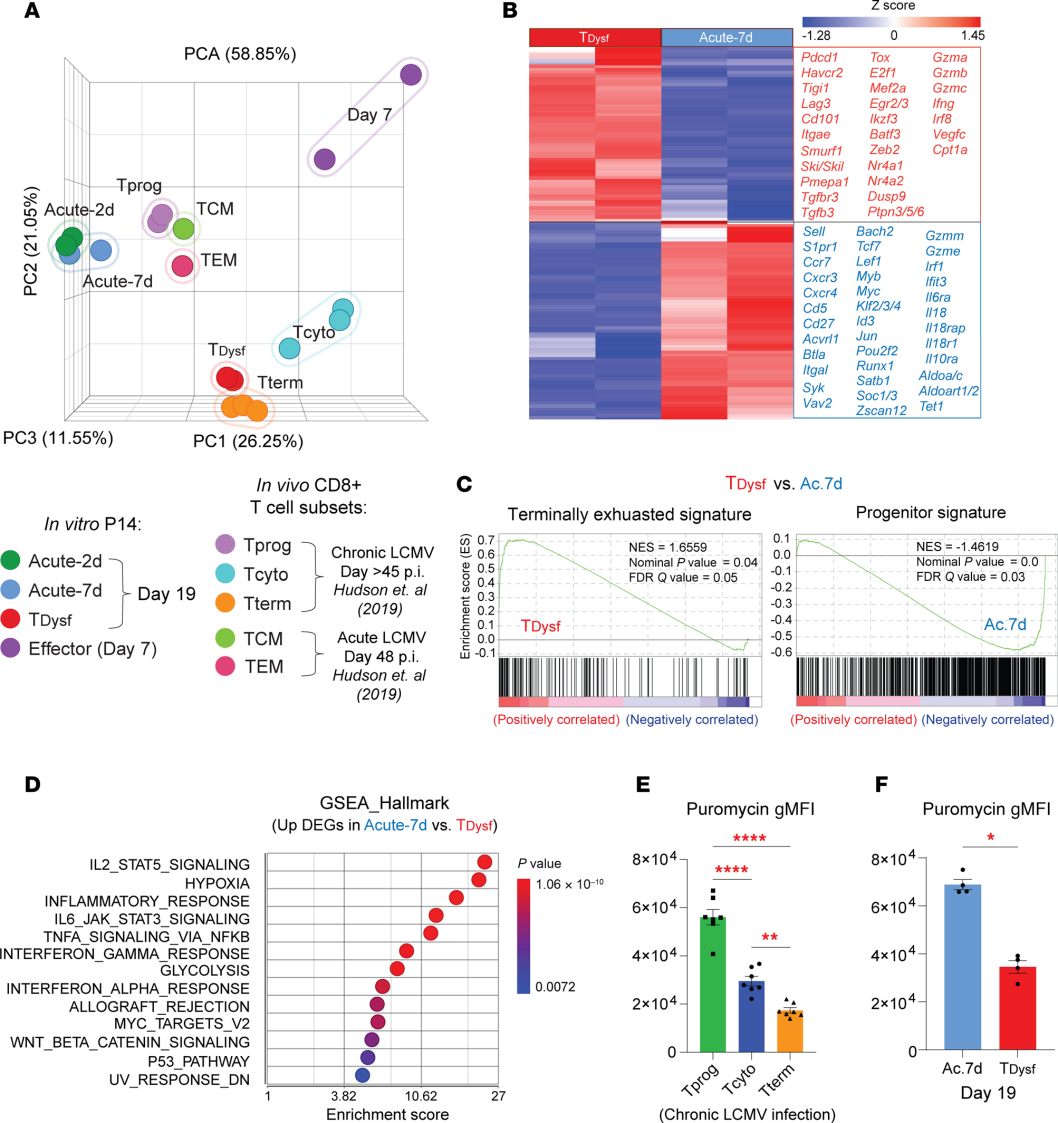
Chronic TCR plus TGF-β1 signals establish the terminal exhaustion transcriptome and impair protein translation. (**A**) Principal component analysis (PCA) plot comparing transcriptional (RNA) signatures for in vitro antigen-stimulated P14 cells (isolated on day 7, “Effector”; or day 19, “T_Dysf_,” “Acute-2d”, “Acute-7d”) to published signatures for PD-1^+^CD8^+^ TEX subsets isolated on day >45 of chronic LCMV infection (progenitor “Tprog” CD101^–^Tim3^–^; cytolytic “T_cyto_” CD101^–^Tim3^+^; terminally exhausted “T_term_” CD101^+^Tim3^+^) (28) or memory CD8^+^ T cell subsets on day 48 postinfection with acute LCMV (central memory “TCM,” effector memory “TEM”) (36). (**B**) Heatmap showing differentially expressed genes (DEGs) between T_Dysf_ and Acute-7d P14 cells. DEGs were plotted based on relative *Z*-score of normalized RNA counts and grouped based on shared expression patterns. (**C**) Gene set enrichment analysis (GSEA) plots for DEGs upregulated in T_Dysf_ versus Acute-7d in comparison to published signatures for TEX subsets on day >45 of chronic LCMV infection (28). (**D**) GSEA score plot compared with Hallmark gene signature for DEGs upregulated in Acute-7d versus T_Dysf_. (**E**) Summary bar graph for intracellular puromycin levels (gMFI) in TEX subsets isolated from LCMV clone 13–infected mice on day 21 postinfection or (**F**) in vitro–stimulated P14 cells on day 19 after GP33 peptide rechallenge. *N* = 2 biological replicates for RNA-Seq, or *n* = 4–6 biological replicates for **E** and **F**, representative of 2 to 3 independent experiments. Statistical significance was determined by (**A**) PCA, (**B**) DESeq2 analysis, or (**D**) GSEA using Partek software or (**C**) using UCSD and Broad Institute GSEA software. Comparisons (**E**) were determined by 1-way ANOVA with Tukey’s multiple comparisons or (**F**) Mann-Whitney *U* test (unpaired, 2-sided). Adjusted *P* value *P < 0.05, **P < 0.01, ****P < 0.0001. Error bars indicate mean ± SEM.

Next, we examined whether in vitro chronic antigen plus TGF-β1 stimulation establishes the transcriptional circuits of T cell exhaustion observed in vivo. We performed RNA-Seq on antigen-experienced P14 cells, FACS-purified on day 7 (Effector stage) or day 19 (Acute-2d, Acute-7d, and T__Dysf__) (Supplemental Figure 5A). Principal component analysis (PCA) revealed that T__Dysf__ cells underwent distinct transcriptional programming and clustered closely with terminally TEX cells from chronic LCMV infection (28) (Figure 3A and Supplemental Figure 5B). In contrast, the transcriptomes of acutely stimulated P14 cells (Acute-2d and Acute-7d) closely resembled those of memory CD8^^+^^ T cells, including TCM and TEM cell subsets from acute LCMV infection and progenitor TEX cells from chronic LCMV infection (28, 36). These results indicate that prolonged TCR and TGF-β1 stimulation accurately models the transcriptional landscape of terminal exhaustion.

T__Dysf__ cells showed significant upregulation of exhaustion-associated genes, including inhibitory receptors shared with Effector cells (*Pdcd1*, *Havcr2*, *Tigit*, *Lag3*), and surface markers and transcription factors (TFs) linked to terminal exhaustion (*Cd101*, *Cd38*, *Tox*, *Nr4a1/2*) (Figure 3B and Supplemental Figure 5C). They also upregulated *Itgae* (encoding CD103, known to be induced by TGF-β1 signals) (37) and negative regulators of TGF-β1 signaling (*Smurf1*, *Ski*, *Skil*, *Pmepa1*) (38). Notably, T__Dysf__ cells retained RNA expression of some effector molecules (*Ifng*, *Gzma*, *Gzmb*, *Gzmk*), though discordance between *Ifng* RNA and protein levels may reflect posttranscriptional regulation, impaired protein synthesis, or the chronic stimulation conditions under which they were isolated, in contrast with acutely stimulated cells, which were resting before transcriptional analysis. Conversely, acutely stimulated cells retained higher expression of stemness- and memory-associated genes (e.g., *Sell*, *Ccr7*, *Tcf7*, *Lef1*, *Cd27*), along with T cell migration– and chemotaxis-linked genes (e.g., *S1pr1*, *Klf2*, *Cxcr3*, *Cxcr4*, *S1pr4*) (Figure 3B and Supplemental Figure 5C).

To further determine whether the upregulated DEGs within T__Dysf__ cells recapitulate the transcriptional signature of TEX cells in vivo, we performed gene set enrichment analysis (GSEA) using progenitor, cytolytic, and terminally TEX subsets from chronic LCMV infection (28). Importantly, T__Dysf__ cells were enriched for the terminally exhausted signature, while Acute-2d and Acute-7d cells closely resembled progenitor TEX cells, and day 7 Effector P14 cells aligned with the cytolytic subset signature (Figure 3C and Supplemental Figure 5, D and E). Comparing single-cell transcriptional signatures of human tumor-infiltrating lymphocytes (TILs) from 21 types of cancer (33), we found that T__Dysf__ cells resembled TEX subsets, including KIR^^+^^TXK^^+^^ NK-like and terminal exhausted TILs (Supplemental Figure 6A), while Acute-7d P14 cells aligned with functional subsets of TILs, closely resembling terminally differentiated effector memory T cell signature (Supplemental Figure 6B). To gain insights into the biological functions of upregulated genes in Acute-7d versus T__Dysf__ cells, we performed Gene Ontology and Hallmark GSEA, which revealed significant enrichment of pathways associated with T cell activation, proliferation, adhesion, IL-2/TNF/IFNG cytokine signaling, and glycolysis in acutely stimulated T cells (Figure 3D and Supplemental Figure 6C). However, upregulated genes in T__Dysf__ cells were enriched for cell cycle–related processes, E2F targets, and G2M checkpoint hallmarks (Supplemental Figure 6, D–G), previously linked with dysfunctional CD8^^+^^ T cells (9, 39). Thus, T__Dysf__ cells transcriptionally resemble terminally TEX cells in chronic infection and cancer.

TEX cells also exhibit impaired protein translation during metabolic stress (40). To evaluate protein translational activity in TEX cells from chronic LCMV infection, we performed a puromycin incorporation assay, in which puromycin is incorporated into nascent polypeptide chains during active protein synthesis (41). Terminally exhausted virus-specific CD8^^+^^ T cells had significantly reduced puromycin incorporation compared with progenitor or cytolytic TEX subsets (Figure 3E). Similarly, T__Dysf__ cells restimulated in vitro on day 19 showed reduced puromycin incorporation compared with Acute-7d cells (Figure 3F and Supplemental Figure 6H). Importantly, puromycin levels positively correlated with Ifnγ and PD-1 protein expression in both groups (Supplemental Figure 6, I and J). Together, these results demonstrate that T__Dysf__ cells acquire the transcriptional features of terminally TEX cells in mice or humans and also impaired protein synthesis, which may explain the discordant RNA-protein expression patterns observed in TEX cells (42).

### *Stable CD8^^+^^ T cell dysfunction requires prolonged stimulation exceeding 2 weeks*.

**Figure 4:**
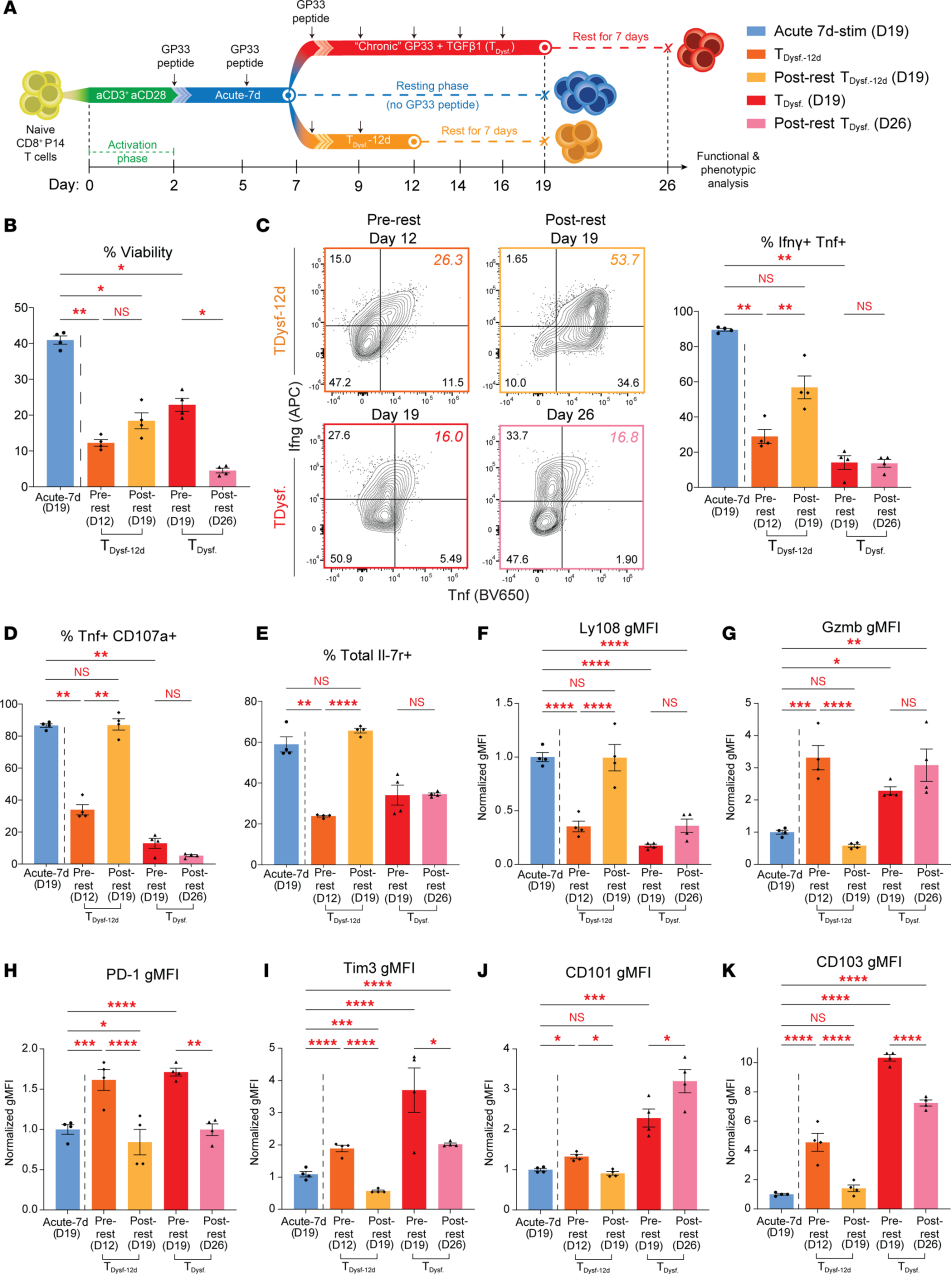
Stable CD8^+^ T cell dysfunction requires prolonged stimulation exceeding 2 weeks. (**A**) Schematic showing resting phase of T_Dysf_ P14 cells under homeostatic conditions. Naive P14 CD8^+^ T cells were stimulated with GP33 peptide and TGF-β1 for either 12 days (T_Dysf_-12d) or 19 days (T_Dysf_), then rested in GP33 and TGF-β1–free medium for 7 days. (**B**) Bar graph depicting frequency of viable P14 cells comparing T_Dysf_-12d pre-rest (day 12) and post-rest (day 19) versus T_Dysf_ pre- and post-rest (day 19 and 26, respectively). (**C** and **D**) Functional assessment of polyfunctional cytokine production. Representative FACS plots and bar graphs showing frequencies of (**C**) Ifnγ^+^Tnf^+^ and (**D**) Tnf^+^CD107a^+^ P14 cells following GP33 peptide rechallenge for P14 CD8^+^ T cells on day 12, 19, and 26 following resting for 7 days. (**E**) Bar graph showing frequency of Total Il7r^+^ P14 cells. (**F**) Expression level (normalized gMFI) of Ly108, (**G**) Gzmb, (**H**) PD-1, (**I**) Tim3, (**J**) CD101, and (**K**) CD103 within P14 cells normalized to Acute-7d on day 19. All *n* = 4 biological replicates, representative of 2 to 3 independent experiments. Adjusted *P* value *P < 0.05, **P < 0.01, ***P < 0.001, ****P < 0.0001. Comparisons were determined by 1-way ANOVA with Tukey’s multiple comparisons (**B**–**K**). Error bars indicate mean ± SEM.

The hallmark of terminal CD8^^+^^ T cell exhaustion is the stability of the dysfunctional state (3, 10). To determine whether T__Dysf__ cells adopt this irreversible phenotype, we performed recovery experiments in which chronically stimulated P14 cells were withdrawn from antigenic stimulation and TGF-β1 signaling at different time points and allowed to rest under homeostatic conditions (Figure 4A). We first tested whether 12 days of chronic stimulation was sufficient to establish stable dysfunction by terminating GP33 and TGF-β1 exposure on day 12, followed by a 7-day rest until day 19 (T__Dysf__-12d condition). T__Dysf__-12d cells demonstrated substantial functional recovery, with restored effector cytokine production and degranulation activity, reflected by increased frequencies of Ifn**γ**^^+^^Tnf^^+^^ and Tnf^^+^^CD107a^^+^^ cells, comparable to acutely stimulated cells (Figure 4, C and D). This was accompanied by improved cell viability (Figure 4B). In contrast, T__Dysf__ cells rested from day 19 to day 26 failed to regain effector function and maintained reduced cytokine and degranulation capacity (Figure 4, C and D). The viability of T__Dysf__ cells also declined throughout the resting period (Figure 4B).

Phenotypic analysis further distinguished reversible from stable dysfunction. T__Dysf__-12d cells downregulated exhaustion markers (PD-1, Tim3, CD101, CD103, and Gzmb) and reexpressed the memory-associated markers Ly108 and Il7r, though CD62L recovery remained modest (Figure 4, E–K, and Supplemental Figure 7, A and B), suggesting a flexible dysfunctional state. In contrast, T__Dysf__ cells rested from day 19 retained high expression of exhaustion markers and failed to reexpress Il7r and Ly108 (Figure 4, E and F, and Supplemental Figure 7A), indicating phenotypic rigidity and stable commitment to terminal exhaustion. To further validate the stability of this state, we extended the recovery period to 11 days of resting (day 19 to day 30). Even after prolonged rest, T__Dysf__ cells failed to restore cytokine production, degranulation, or expression of memory markers, while exhaustion markers remained elevated (Supplemental Figure 7, C–K). These findings reveal a threshold in stimulation duration for stable exhaustion programming. While less than 2 weeks of chronic stimulation results in reversible functional impairment, extending stimulation to about 3 weeks establishes irreversible reprogramming characteristic of terminal dysfunction. The inability of T__Dysf__ cells to recover function or memory traits, even after prolonged rest, validates our in vitro model as a faithful representation of the irreversible T cell exhaustion observed in vivo.

### *Dysfunctional P14 cells recapitulate the heterogeneity of exhausted CD8^^+^^ T cells generated in vivo*.

**Figure 5:**
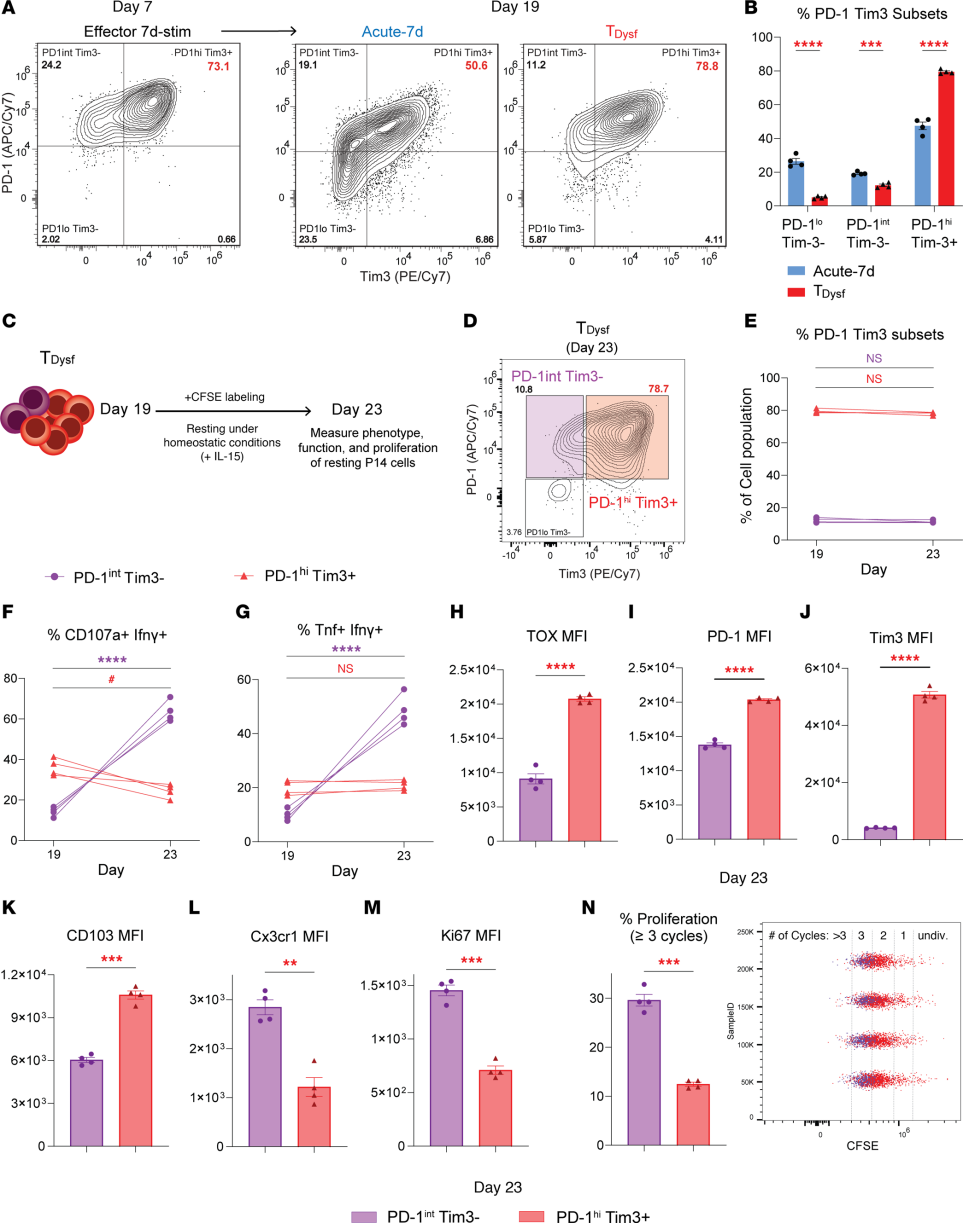
Dysfunctional CD8^+^ T cells recapitulate the heterogeneity of exhausted CD8^+^ T cells generated in vivo. (**A**) Representative FACS plots of PD-1 and Tim3 expression for P14 CD8^+^ T cells on both day 7 (Effector 7d-stim) and day 19 (Acute-7d or Chronic GP33^+^TGF-β1: T_Dysf_) after rechallenging with GP33 peptide. (**B**) Summary bar graph showing % of PD-1– and Tim3-expressing subsets on P14 CD8^+^ T cells on day 19. (**C**) Schematic for the resting phase of T_Dysf_ P14 cells under homeostatic conditions. (**D**) Representative FACS plot of PD-1 and Tim3 expression for P14 CD8^+^ T cells on day 23 following resting for 4 days. (**E**) Longitudinal tracking on day 19 and 23 of the frequency of PD-1^int^Tim3^–^ (purple) and PD-1^hi^Tim3^+^ (red) P14 cells, (**F**) frequency of CD107a^+^Ifnγ^+^ P14 cells, and (**G**) frequency of Tnf^+^Ifnγ^+^ P14 cells within PD-1^int^Tim3^–^ and PD-1^hi^Tim3^+^ subsets. (**H**) Expression levels (gMFI) of TOX, (**I**) PD-1, (**J**) Tim3, (**K**) CD103, (**L**) Cx3cr1, and (**M**) Ki67 within PD-1^int^Tim3^–^ and PD-1^hi^Tim3^+^ subsets on day 23. (**N**) Bar graph showing frequencies of divided P14 cells with ≥3 proliferation cycles by CFSE staining. All *n* = 4 biological replicates, representative of 2 to 3 independent experiments. Adjusted *P* value ^#^*P* < 0.05 for PD-1^hi^Tim3^+^ cells (**F**), **P < 0.01, ***P < 0.001, ****P < 0.0001. Comparisons were determined by 1-way ANOVA (**B**), 2-way ANOVA (**E**–**G**), or Mann-Whitney *U* test (unpaired, 2-sided) (**H**–**N**). Error bars indicate mean ± SEM.

T cell dysfunction is a gradual process resulting in heterogeneous populations. On day 19, we identified distinct subsets: a dominant PD-1^^hi^^Tim3^^+^^ population and a minor PD-1^^int^^Tim3^^–^^ subset, suggesting that heterogeneity arises during chronic stimulation (Figure 5, A and B, and Supplemental Figure 8A). Resting Acute-7d P14 cells downregulated PD-1 and Tim3, leading to the emergence of a PD-1^^lo^^Tim3^^–^^ subset (Figure 5A). Across all these subsets, acutely stimulated P14 cells maintained significantly higher expression levels of effector cytokines and memory markers (Ifnγ, Tnf, CD107a, Ly108, Il7r, and CD62L) compared with T__Dysf__ cells (Supplemental Figure 8, B–D and H). Remarkably, the PD-1^^hi^^Tim3^^+^^ T__Dysf__ subset exhibited reduced Perforin and Ki67 (Supplemental Figure 8, F and G), while maintaining heightened expression of Tox (43) (Supplemental Figure 8E), consistent with terminal dysfunction.

To test stability of T__Dysf__ P14 subsets, we CFSE-labeled and rested T__Dysf__ cells for 4 days (day 19–23, Figure 5C). The PD-1^^hi^^Tim3^^+^^ subset remained severely dysfunctional with high expression of inhibitory receptors and Tox (Figure 5, D–J, and Supplemental Figure 8, I–L). In contrast, PD-1^^int^^Tim3^^–^^ cells recovered polyfunctionality and expressed higher levels of Cx3cr1, a marker of the cytolytic TEX subset, while PD-1^^hi^^Tim3^^+^^ cells retained CD103 expression (Figure 5, K and L). PD-1^^int^^Tim3^^–^^ cells also showed greater homeostatic proliferation, with higher proportions undergoing at least 3 division cycles during rest (Figure 5, M and N). Together, these findings show that 3 weeks of chronic stimulation drives stable CD8^^+^^ T cell dysfunction, with the dominant PD-1^^hi^^Tim3^^+^^ subset exhibiting features of terminal exhaustion.

### *Terminal dysfunction of in vitro chronically stimulated CD8^^+^^ T cells is stabilized by exhaustion-specific epigenetic programs*.

**Figure 6:**
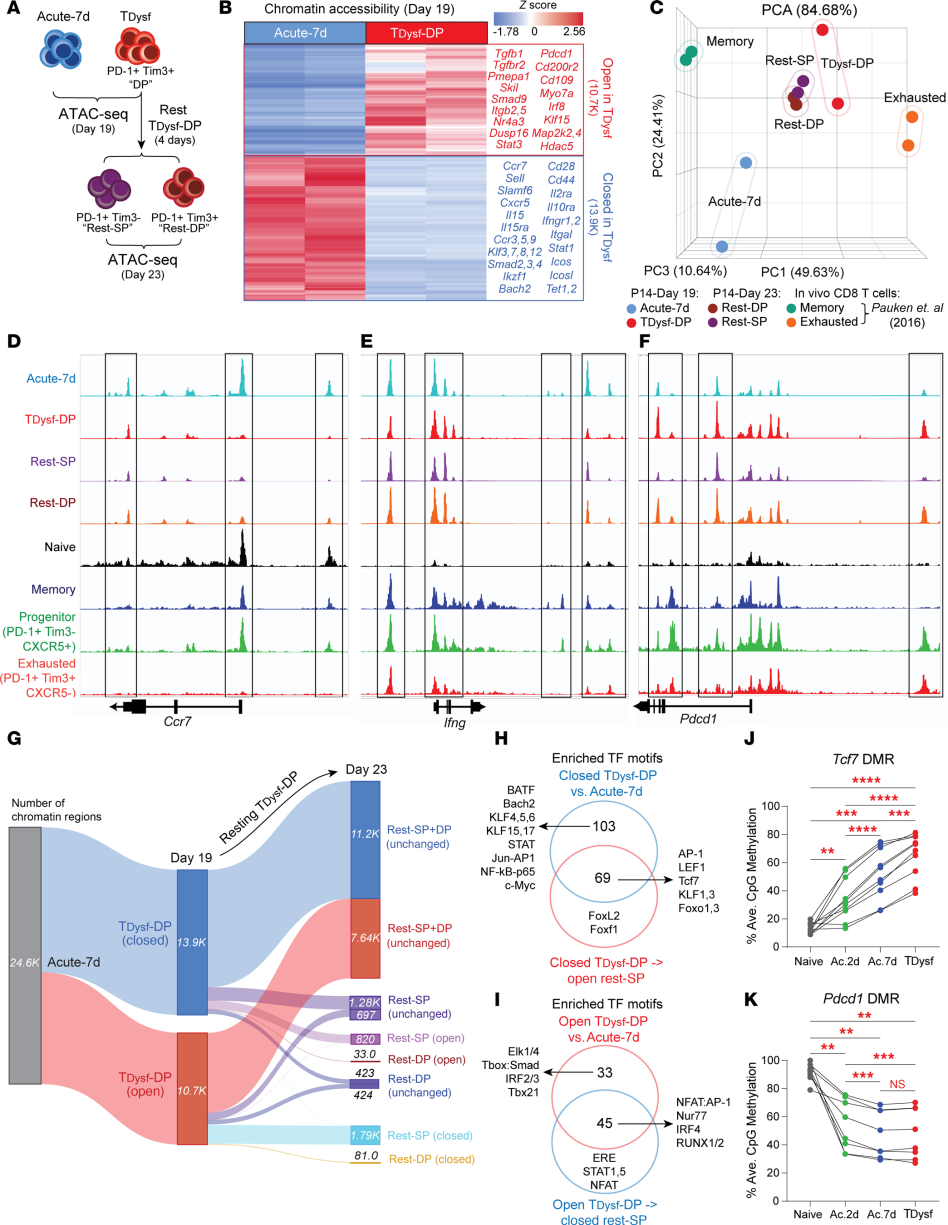
Terminal dysfunction of in vitro–generated CD8^+^ T cells is stabilized by exhaustion-associated epigenetic programs. (**A**) Schematic for ATAC-sequencing of P14 cells from the models described in Figure 2A and Figure 5C. T_Dysf_ cells were FACS-sorted on day 19 of chronic stimulation as the PD-1^+^Tim3^+^ (double-positive, “T_Dysf_-DP”) and rested for 4 days. Rested T_Dysf_-DP cells were sorted on day 23 as PD-1^+^Tim3^–^ (single-positive, “Rest-SP”) or PD-1^+^Tim3^+^ (double-positive, “Rest-DP”). (**B**) Heatmap showing differentially open chromatin regions (OCRs) between Acute-7d and T_Dysf_-DP cells (day 19) plotted based on relative *Z*-score with example genes listed. Red indicates “open” and blue indicates “closed” regions. (**C**) PCA comparing chromatin accessibility profiles for in vitro Acute-7d or T_Dysf_ subsets to published signatures for in vivo memory or exhausted CD8^+^ T cells (23). (**D**) Representative snapshots of accessible chromatin peaks (mapped in Integrative Genomics Viewer, IGV) within in vitro P14 or in vivo naive, memory, progenitor, or exhausted CD8^+^ T cells (44) at gene loci for *Ccr7*, (**E**) *Ifng*, and (**F**) *Pdcd1*. (**G**) Alluvial plot tracking the number of chromatin regions from Acute-7d cells that became open/closed in T_Dysf_-DP (day 19) compared with Acute-7d and regions from T_Dysf_-DP that remained unchanged or became open/closed in rested T_Dysf_ subsets (day 23). (**H**) Venn diagram comparing transcription factor (TF) motifs enriched within closed OCRs in T_Dysf_-DP cells and regions that became open in Rest-SP cells or (**I**) open OCRs in T_Dysf_-DP cells and regions that became closed in Rest-SP cells. (**J**) Average % CpG methylation at *Tcf7* and (**K**) *Pdcd1* differentially methylated regions (DMRs) within naive or in vitro–generated P14 cells (day 19). *N* = 2 biological replicates, representative of 2 to 3 independent experiments. Statistical significance was determined by DESeq2 (**B** and **G**) or PCA (**C**) using Partek software. Comparisons in **J** and **K** were determined by repeated measures 1-way ANOVA with Tukey’s multiple comparisons. Adjusted *P* value **P < 0.01, ***P < 0.001, ****P < 0.0001.

T cell exhaustion is marked by distinct epigenetic changes that lock terminally exhausted CD8^^+^^ T cells into a stable dysfunctional state (3, 7–14). This remodeling involves loss of chromatin accessibility at effector function–, stemness-, and memory-associated genes while maintaining accessibility at exhaustion-linked loci (11, 23, 44). To define these changes within our in vitro–generated P14 cells, we performed ATAC-Seq on Acute-7d and T__Dysf__ (PD-1^^hi^^Tim3^^+^^) P14 cells isolated on day 19 (Figure 6A). T__Dysf__ cells acquired a distinct open chromatin landscape, with over 24,600 differentially open chromatin regions (OCRs; *P* < 0.01, fold change ≥ 2), including ~13,900 regions with reduced accessibility and ~10,700 regions with increased accessibility relative to Acute-7d cells (Figure 6B). These OCRs were mostly intronic and intergenic (Supplemental Figure 9, A and B), suggesting regulatory roles. Regions with increased accessibility in T__Dysf__ cells included TGF-β1 signaling genes (e.g., *Tgfb1*, *Tgfbr2*, *Pmepa1*, *Skil*, *Smad9*), inhibitory receptors (e.g., *Pdcd1*, *Cd200r2*), MAPK pathway genes (e.g., *Dusp16*, *Map2k2*, *Map2k4*), and exhaustion-associated TFs (*Nr4a3*, *Stat3*, *Irf8*) (45, 46) (Figure 6B). In contrast, decreased-accessibility regions included stemness/memory genes (e.g., *Ccr7*, *Sell*, *Slamf6*, *Cxcr5*, and *Il15ra*) and effector/TCR signaling genes (e.g., *Cd28*, *Cd44*, *Il2ra*, *Icos*, *Icosl*, *Itgal*, *Ifngr1*, and *Ifngr2*) (2, 29, 47) (Figure 6B). Pathway enrichment analysis showed that closed OCRs in T__Dysf__ cells were enriched for IL-2/STAT5, TCR signaling, Ifnγ, and inflammatory response pathways, while open OCRs were enriched for PI3K/AKT/mTOR, Hedgehog and Notch signaling, and GTPase activity pathways (Supplemental Figure 9, C–F). These results demonstrate extensive epigenetic remodeling in T__Dysf__ cells, consistent with a stable, epigenetically reprogrammed dysfunctional state.

To determine whether this epigenetic landscape mirrors in vivo exhaustion-specific programming, we compared ATAC-Seq profiles of T__Dysf__ and Acute-7d P14 cells to virus-specific CD8^^+^^ T cells from acute and chronic LCMV infections (23). PCA of the top 5,000 most variable peaks showed that T__Dysf__ cells clustered with terminally exhausted cells, while Acute-7d cells aligned more with memory CD8^^+^^ T cells (Figure 6C). Importantly, OCRs between T__Dysf__ and Acute-7d cells reflected patterns observed in terminally exhausted (PD-1^^+^^Tim3^^+^^CXCR5^^–^^) versus progenitor (PD-1^^+^^Tim3^^–^^CXCR5^^+^^) TEX cells or memory CD8^^+^^ T cells (44). For instance, T__Dysf__ cells exhibited similarly reduced accessibility at memory/progenitor TEX-associated genes (e.g., *Ccr7*, *Ifng*, *Tnf*, *Cxcr5*, *Sell*, *Slamf6*, *Il7r*, *Tcf7*) and increased accessibility at terminal exhaustion genes (e.g., *Pdcd1*, *Havcr2*, *Cd244a*) (Figure 6, D–F, and Supplemental Figure 9, I–Q). Thus, T__Dysf__ cells undergo exhaustion-specific chromatin remodeling consistent with terminally TEX cells in vivo, while Acute-7d cells retain a memory-like open chromatin landscape.

To assess stability after antigen stimulation withdrawal, we performed ATAC-Seq on PD-1^^hi^^Tim3^^+^^ cells (“double-positive”; Rest-DP) and PD-1^^int^^Tim3^^–^^ (“single-positive”; Rest-SP) P14 cells isolated on day 23 following withdrawal from chronic stimulation (Figure 5, C and D, and Figure 6A). Both populations retained the chromatin accessibility profiles of T__Dysf__ and terminally TEX cells (Figure 6, C–F, and Supplemental Figure 9, I–Q), with ~92.8% of closed and ~81.9% of open T__Dysf__-specific OCRs unchanged (Figure 6G and Supplemental Figure 10, A and B), indicating strong epigenetic stability. However, Rest-SP cells showed partial reversal in a subset of regions: ~5.9% (820 OCRs) of closed regions regained accessibility, including *Cd44*, *Sell*, *Btla*, and *Icos*; TFs (*Izkf1*, *Ikzf3*, *Bach2*, *Stat4*, *Zeb2*, *Klf12*, and *Foxo3*); and cytokine receptors (*Il12rb2*, *Il18r1*) (Figure 6G and Supplemental Figure 10, A–C). Conversely, ~16.7% (~1,790 OCRs) of open regions became less accessible, including peaks at TFs (e.g., *Bcl2*, *Tox*, *Irf4*, *Prdm2*, and *Foxp1*), histone-modifying enzymes (e.g., *Ezh2* and *Hdac5*), and signaling molecules (e.g., *Mapk10*, *Wnt3*, *Wnt11*, *Notch4*, and *Tbk1*) (Figure 6G and Supplemental Figure 10, A–C). Reopened regions in Rest-SP cells were enriched for IL-2 and TCR signaling pathways, while regions that lost accessibility were enriched for GTPase activity and MAPK signaling pathways (Supplemental Figure 9, G and H). These results demonstrate that T__Dysf__ cells retain a largely stable, epigenetically “scarred” chromatin state after removal of chronic signals — particularly in the PD-1^^hi^^Tim3^^+^^ cell population — closely resembling the fixed epigenetic profile of terminally TEX cells in vivo (11, 23, 44).

To gain insights into mechanisms underlying this stability, we performed TF motif enrichment analysis on T__Dysf__-specific OCRs that remained unchanged following resting. Stable closed OCRs were enriched for motifs of TFs regulating effector and memory programs (e.g., BATF, Bach2, AP1, NF-κB, c-Myc, KLF, and STATs), while reopened peaks in Rest-SP cells were enriched for Tcf7, LEF1, KLF1/3, and Foxo1/3 motifs (Figure 6H), suggesting limited reengagement of memory-regulatory TFs. Conversely, stable open OCRs in Rest-SP and Rest-DP cells were enriched for exhaustion-linked motifs (e.g., Elk1/4, Smad, IRF2/3, and Tbx21), while Rest-SP cells exhibited reduced accessibility at OCRs containing NFAT, Nur77, IRF4, RUNX1/2, and STAT1/5 motifs, suggesting partial rewiring of TCR and cytokine-dependent signaling (Figure 6I). Overall, these findings reveal that specific TF networks preserve the dysfunctional epigenetic state by maintaining accessibility at exhaustion-related motifs and closing memory- and effector-regulatory regions, limiting recovery even after stimulation withdrawal.

Our previous work demonstrated that de novo DNA methylation drives epigenetic silencing of memory and effector programs within TEX cells (8, 9). Additionally, specific DNA demethylation changes at the *Pdcd1* locus promote PD-1 upregulation in TEX cells, beginning in the effector phase of CD8^^+^^ T cell differentiation (26, 48). To determine whether T__Dysf__ cells acquire distinct DNA methylation programs associated with stable exhaustion, we performed targeted DNA methylation analysis at key differentially methylated genomic regions (DMRs) (8) (Supplemental Figure 11, A–C). Our analysis revealed that T__Dysf__ cells acquired substantial de novo DNA methylation at both *Tcf7* and *Ccr7* loci, consistent with downregulation of Tcf1 and Ccr7 proteins (Figure 6J and Supplemental Figure 11D), while acutely stimulated cells recovered both molecules by day 19 (Supplemental Figure 11, E–I). In contrast, the *Pdcd1* DMR was demethylated in both groups (Figure 6K), consistent with DNA demethylation at *Pdcd1* during early effector differentiation (26). However, PD-1 remained higher on T__Dysf__ cells on day 19, likely due to sustained antigen exposure (26, 27, 49) (Supplemental Figure 11, E–G). Thus, acquisition of de novo DNA methylation at stemness and memory-associated loci limits recovery, paralleling epigenetic constraints in these programs within terminally exhausted CD8^^+^^ T cells in vivo.

### *Dysfunction of in vitro–generated T__Dysf__ cells is irreversible during in vivo rechallenge*.

**Figure 7:**
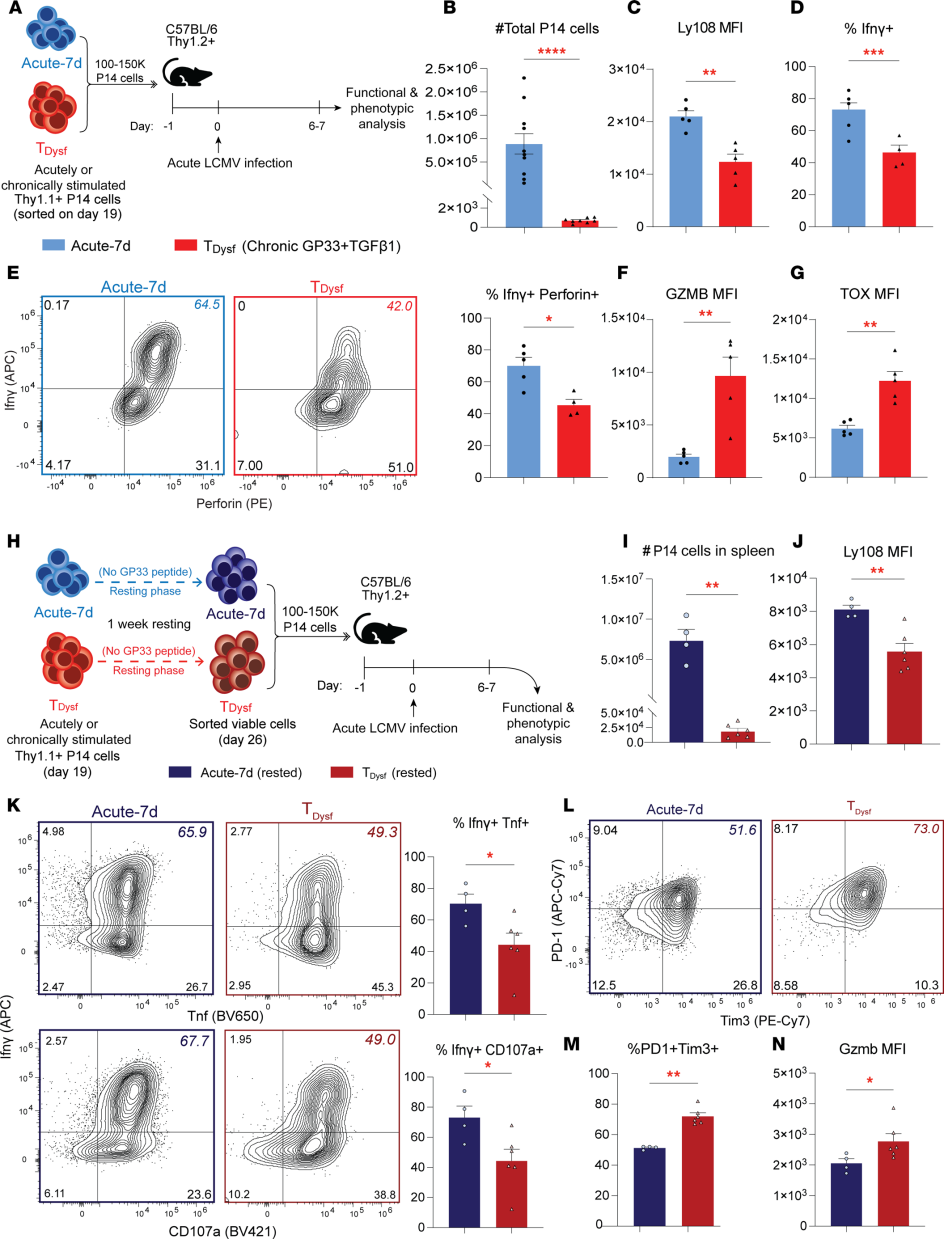
Dysfunction of in vitro–generated T_Dysf_ cells is irreversible during in vivo rechallenge. (**A**) Schematic for adoptive transfer of “Acute-7d” (blue) or “T_Dysf_” (red) P14 cells into congenically distinct C57BL/6 mice on day 19, followed by acute LCMV infection and analysis on day 6–7 postinfection. (**B**) Total P14 cell numbers from spleens, livers, and lungs. Panels **C**–**G** and **I**–**N** are for spleens. (**C**) Bar graph showing expression level (gMFI) of Ly108 or (**D**) % Ifnγ^+^ P14 cells after ex vivo GP33 peptide rechallenge. (**E**) Representative FACS plots showing Ifnγ and Perforin expression and summary bar graph showing % Ifnγ^+^Perforin^+^ P14 cells. (**F**) Bar graph showing expression level (gMFI) of Gzmb or (**G**) TOX. (**H**) Schematic for adoptive transfer of “Rested Acute-7d” (dark blue) or “Rested T_Dysf_” (dark red) P14 cells on day 26 into congenically distinct C57BL/6 mice, followed by acute LCMV infection and analysis on day 6–7 postinfection. (**I**) Bar graph showing total number of P14 cells isolated from spleens or (**J**) expression level (gMFI) of Ly108. (**K**) Representative FACS plots showing Ifnγ and Tnf or CD107a expression and summary bar graph showing % of Ifnγ^+^Tnf^+^ or Ifnγ^+^CD107a^+^ P14 cells. (**L**) Representative FACS plots showing PD-1 and Tim3 expression and (**M**) summary bar graph showing % of PD-1^+^Tim3^+^ P14 cells. (**N**) Bar graph showing the expression level (gMFI) of Gzmb. For all FACS plots (**E**, **K**, and **L**), Acute-7d plots are representative of 1 recipient mouse, while the T_Dysf_ plots show data combined from all T_Dysf_ recipient mice. For **B**, data were pooled from 2 independent experiments with *n* = 3–5 biological replicates per group for each experiment. For all other panels, *n* = 4–6 biological replicates from 2 to 3 independent experiments. Adjusted *P* value *P < 0.05, **P < 0.01, ***P < 0.001, ****P < 0.0001. Comparisons were determined by Mann-Whitney *U* test (unpaired, 2-sided). Error bars indicate mean ± SEM.

Finally, we assessed the in vivo recall responses of our in vitro–generated T__Dysf__ cells, as a key hallmark of T cell exhaustion is the compromised ability to respond after antigen rechallenge (11, 24). We sorted congenically distinct Thy1.1^^+^^ P14 cells from T__Dysf__ or Acute-7d groups on day 19 and adoptively transferred them into naive C57BL/6 animals. One day later, mice were challenged with acute LCMV infection, and P14 recall responses were assessed after 6–7 days (Figure 7A). While Acute-7d cells underwent extensive proliferation, T__Dysf__ cells showed impaired expansion (Figure 7B), as well as sustained Ly108 downregulation (Figure 7C). Upon ex vivo GP33 peptide restimulation, T__Dysf__ cells exhibited reduced effector functions (Ifnγ and Perforin), while Acute-7d cells maintained heightened polyfunctionality (Figure 7, D and E). T__Dysf__ cells also expressed higher levels of exhaustion-associated molecules, such as Tox and Gzmb, in spleen, liver, and lungs (Figure 7, F and G, and Supplemental Figure 12, A and B). To test whether resting T__Dysf__ cells improves their recall capacity, we adoptively transferred T__Dysf__ P14 cells after a 7-day rest period, followed by acute LCMV infection (Figure 7H). Despite rest, T__Dysf__ cells remained impaired in proliferation and expression of Ly108 and Il7r (Figure 7, I and J, and Supplemental Figure 12C), showed diminished effector function (e.g., Ifnγ, Tnf, and CD107a) (Figure 7K), and retained high levels of exhaustion markers (PD-1, Tim3, Gzmb, and Tox) (Figure 7, L–N, Supplemental Figure 12, D–F). Together, these findings demonstrate that ~3 weeks of in vitro chronic stimulation drives CD8^^+^^ T cells into a stable, terminally dysfunctional program that mirrors in vivo terminal exhaustion, including severely impaired recall capacity.

## Discussion

Epigenetic scarring of T cell exhaustion programs is a major cell-intrinsic barrier to T cell–based immunotherapies. Faithful model systems for T cell exhaustion are essential for understanding the individual and cooperative contributions of factors driving the epigenetic progression toward terminal exhaustion. While common animal models of T cell exhaustion, such as chronic LCMV infection or tumor models, are among the most reliable systems for these investigations, they present inherent complexities that make it challenging to dissect the precise contributions of individual factors to the epigenetic programming of TEX cells. Therefore, there is a major need to develop reliable reductionist approaches that enable precise control and isolation of individual signals, facilitating a deeper understanding of how specific factors influence the programming events underlying T cell exhaustion.

Recent studies have introduced new in vitro models of T cell dysfunction through repeated polyclonal anti-CD3 or antigen-specific stimulation of murine CD8^^+^^ T cells (18–21). However, a key limitation has been the poor survival and widespread cell death of repeatedly TCR-stimulated CD8^^+^^ T cells, preventing chronic stimulation from extending beyond 5–10 days (20). This restricts the ability to replicate in vivo settings, such as chronic viral infections, where CD8^^+^^ T cells experience prolonged stimulation over ~3 weeks before developing a terminally dysfunctional state (2, 24). Indeed, we found that ~1–2 weeks of repeated stimulation induces some exhaustion-like features, marked by increased coexpression of inhibitory receptors; however, these conditions fail to establish epigenetically stable terminal dysfunction. Instead, after acute antigen stimulation is removed, CD8^^+^^ T cells rapidly recover effector functions and memory programs. These findings align with the demonstrated plasticity of day 8–15 effector CD8^^+^^ T cells, which can regain function and memory potential when removed from chronic LCMV-infected environments and transferred to antigen-free conditions (24). Thus, short-term antigen stimulation alone is insufficient for inducing terminal dysfunction; instead, it drives effector differentiation with preserved memory potential, mimicking CD8^^+^^ T cell responses during acute viral infections, during which viruses are typically cleared within 1–2 weeks (50).

Here, we have developed a faithful animal cell model for terminal exhaustion by delivering chronic TGF-β1 signals between days 7 and 19 to chronically antigen-stimulated P14 CD8^^+^^ T cells. This in vitro model builds on our previous work showing that chronic, post-effector TGF-β1 signaling is necessary for driving terminal dysfunction in chronically TCR-stimulated human CD8^^+^^ T cells (9). Here, we demonstrate that prolonged TGF-β1 signals are critical for maintaining the survival of chronically antigen-stimulated P14 (T__Dysf__) cells while also promoting an epigenetically stabilized, terminally dysfunctional state. Under these conditions, T__Dysf__ cells exhibited the full range of phenotypic, functional, transcriptional, epigenetic, and metabolic characteristics of terminal exhaustion. The stability of this terminal exhaustion program was validated by resting T__Dysf__ cells following antigen withdrawal, which revealed persistent epigenetic “scarring” of the dysfunctional state. A limited degree of epigenetic flexibility was observed only within the minor PD-1^^int^^Tim3^^–^^ subset that emerged after resting T__Dysf__ cells. Furthermore, adoptive transfer studies of T__Dysf__ cells — both before and after resting — demonstrated that T__Dysf__ cells remained terminally dysfunctional, as they failed to regain effector functions or proliferate in response to acute LCMV infection.

Our tractable model of T cell exhaustion described here offers several advantages for studying molecular mechanisms that underline terminal dysfunction. Specifically, the ex vivo culture model (a) enables tracking of the developmental trajectory of T cell exhaustion over extended periods; (b) facilitates mechanistic studies of various signals from infected tissue or tumor microenvironments; (c) supports high-throughput molecular investigations of terminal T cell exhaustion, improving both the technical and economic efficiency, compared with genetic or therapeutic screening studies performed in vivo; (d) provides a more reliable tool for identifying key factors that stabilize the dysfunctional state in CD8^^+^^ T cells; and (e) offers physiologically relevant conditions for studying antigen-specific T cell responses by using antigen-dependent stimulation, rather than polyclonal, CD3-dependent stimulation. The T__Dysf__ model can be leveraged to design a discovery platform for the development of more effective immunotherapies aimed at restoring the function of severely dysfunctional T cells, ultimately enhancing treatment outcomes for patients with cancer or chronic infections.

## Methods

### *Sex as a biological variable*.

Our study was designed by accounting for sex as a biological variable. We examined both male and female animals, and similar findings are reported for both sexes.

### *Animals*.

All mice used in the LCMV infection experiments from this study were housed at The Ohio State University animal facilities under the IACUC-approved guidelines. This facility included an ambient temperature kept in a range of 68°C–76°C, relative humidity 30%–70%, and a 12-hour dark/12-hour light cycle (light 6 am to 6 pm). Mice were fed standard Teklad 7912 chow (Envigo). Transgenic mice expressing a TCR specific for the LCMV peptide GP33 (P14 donor mice) were bred in-house at The Ohio State University on a C57BL/6 background. Recipient C57BL/6 mice were purchased from The Jackson Laboratory and used at approximately 8 weeks of age. Recipient mice were sex-matched with donor mice.

### *In vitro chronic stimulation of mouse P14 CD8^^+^^ T cells*.

On day 0 of the in vitro chronic stimulation model, CD8^^+^^ T cells were isolated from spleens of healthy, uninfected male or female P14 transgenic mice (≥8 weeks old). Spleens were mashed through a 100 μm filter and subjected to RBC lysis. Splenocytes were enriched for CD8^^+^^ T cells using the EasySep Mouse CD8^^+^^ T Cell Isolation Kit (StemCell Technologies), then stained for spectral cytometry to confirm purity, viability, and GP33 tetramer specificity. CD8^^+^^ T cells were seeded into 6-well, flat-bottom, tissue culture–treated plates coated with mouse purified anti-CD3 (5 μg/mL; clone: 17A2; catalog 100102) and anti-CD28 (10 μg/mL; clone: 37.51; catalog 02102) (both from BioLegend), at a density of 1 × 10^^6^^ to 3 × 10^^6^^ CD8^^+^^ T cells/well in 3 mL of complete RPMI medium (10% fetal bovine serum and 1× Penicillin/Streptomycin from Gibco, 200 IU/mL of recombinant human IL-2 from PeproTech, and 50 μM of 2-Mercaptoethanol from MilliporeSigma) to stabilize IL-2 activity and CD8^^+^^ T cell priming (51). Plates were incubated at 37°C and 5% CO__2__ for 2 days to allow T cell activation.

On day 2, activated P14 CD8^^+^^ T cells were pooled, washed, and resuspended in T cell media (complete RPMI medium containing 20 IU/mL of recombinant human IL-2 and 10 ng/mL of recombinant human IL-15 from PeproTech). Cells were seeded into 96-well, U-bottom plates at 1 × 10^^5^^ to 2 × 10^^5^^ viable cells/well. For the 2d-stimulated condition, cells were cultured without GP33 peptide. For Acute-7d and Chronic groups, GP33 peptide (0.3 μg/mL) was added. On day 7, Acute-7d cells were maintained in peptide-free T cell media. For the Chronic + TGF-β1 T__Dysf__ condition, recombinant human TGF-β1 (5 ng/mL; PeproTech) was added to the GP33 peptide–containing medium beginning on day 7. Anti-CD28 was omitted during the chronic stimulation phase to simulate the declining costimulatory environment during chronic viral infections and tumors, where CD28 expression is progressively lost as TEX cells become terminally exhausted. Media were replenished every 2–3 days with fresh GP33 peptide and TGF-β1 until day 19.

On day 19 of chronic stimulation, P14 cells were sorted for viable CD8^^+^^ T cells (Sony MA900 Cell Sorter) and used for adoptive transfer, in vitro recovery experiments, or ATAC-Seq or snap-frozen at –80°C to use for RNA and DNA methylation sequencing analyses.

For recovery experiments, sorted P14 CD8^^+^^ T cells from day 12 or 19 of chronic stimulation were seeded at 5 × 10^^4^^ cells/well in complete RPMI medium containing IL-15 (10 ng/mL). Cells were cultured for 4–12 days and analyzed on days 23, 26, or 30 of in vitro culture.

### *Plate-bound in vitro stimulation of mouse P14 CD8^^+^^ T cells*.

Following a 2-day activation phase with mouse purified anti-CD3 (5 μg/mL) and anti-CD28 (10 μg/mL) (BioLegend), activated P14 CD8^^+^^ T cells were resuspended in complete RPMI medium containing 20 IU/mL of recombinant human IL-2 and 10 ng/mL of recombinant human IL-15 (PeproTech). P14 cells were then seeded into tissue culture–treated, 96-well, flat-bottom plates coated with anti-CD3 (5 μg/mL) at a density of 1 × 10^^5^^ to 2 × 10^^5^^ viable cells per well. Cells were transferred to freshly coated anti-CD3 plates every 2 days and analyzed on day 5 and 9.

### *Functional analysis of in vitro–stimulated P14 CD8^^+^^ T cells*.

The effector function and cytokine production of in vitro–stimulated P14 cells were analyzed on days 0, 2, 5, 7, 12, 19, 23, 26, and 30 after initial activation. Cells were collected at these time points and stimulated for either 3 hours with PMA/ionomycin plus protein transport inhibitors (eBioscience Cell Stimulation Cocktail, Thermo Fisher Scientific) or for 5 hours with GP33 peptide (0.3 μg/mL) plus protein transport inhibitors (eBioscience Brefeldin A and Monesin, Thermo Fisher Scientific) at 37°C. A spectral cytometry antibody targeting mouse CD107a (Brilliant Violet 421, BioLegend) was added at the beginning of each stimulation to track CD8^^+^^ T cell degranulation activity. Stimulation was immediately followed by antibody staining for spectral cytometry analysis.

### *Puromycin incorporation assay*.

For puromycin incorporation analysis (to measure protein translation capacity), P14 cells were stimulated for 5 hours with GP33 peptide (0.3 μg/mL) plus protein transport inhibitors as above. During the final 30 minutes of incubation, puromycin was added to the media at a concentration of 10 μg/mL (MilliporeSigma). During intracellular staining following stimulation, anti-puromycin antibody (Alexa Fluor 488, BioLegend) was used to label and measure puromycin uptake by spectral cytometry.

### *Adoptive transfer and in vivo rechallenge of in vitro–stimulated P14 CD8^^+^^ T cells*.

For adoptive transfer experiments, antigen-experienced P14 cells were sorted on day 19 or on day 26 (following 7 days of rest) of the in vitro stimulation protocol to isolate the viable CD8^^+^^ T cells using the Sony MA900 Cell Sorter. Immediately following sorting, P14 cells from the 3 described conditions (Acute-2d, Acute-7d, and T__Dysf__) were adoptively transferred into wild-type, congenically distinct (Thy1.1^^–^^Thy1.2^^+^^) C57BL/6 mice (male or female depending on P14 cell identity, age-matched at ≥8 weeks old) at 100,000–150,000 P14 cells/mouse from 1 of the 3 conditions via i.v. retro-orbital injection. One day following adoptive transfer, recipient mice were infected with LCMV Armstrong virus (2 × 10^^5^^ PFU/mouse) via i.p. injection. Mice were euthanized on day 6–7 postinfection, and lymphoid (spleens) and peripheral tissues (livers and lungs) were harvested and processed into single-cell suspensions as previously described (9). Tissue samples were then used for ex vivo GP33 peptide stimulation for 5 hours followed by spectral cytometry staining to assess the function and phenotype of the transferred P14 cells.

### *Spectral cytometry antibody staining*.

Single-cell suspensions were stained with a comprehensive panel, and data were collected using a Cytek Aurora 4-laser spectral cytometer and SpectroFlow software (version 3.3). Dead cells were stained with Ghost Dye Violet 510 (catalog 50-105-2992, Tonbo Biosciences, 1:600 dilution). Mouse surface antibodies used for staining throughout this study include Brilliant Violet 421 anti-CD107a (LAMP-1; clone 1D4B, 1:150 dilution; catalog 121617), Brilliant Violet 605 anti-CD62L (MEL-14, 1:400 dilution; catalog 104437), Brilliant Violet 605 anti-CD103 (2E7, 1:400 dilution; catalog 121433), Brilliant Violet 650 anti-Ccr7 (4B12, dilution 1:200; catalog 120137), Brilliant Violet 711 anti-Cx3cr1 (SA011F11, 1:300 dilution; catalog 149031), Pacific Blue anti-Ly108 (SLAMF6; 330-AJ, 1:150 dilution; catalog 134608), PerCP/Cyanine5.5 anti-CD8a (53–6.7, 1:300 dilution; catalog 100734), Alexa Fluor 700 anti-CD44 (IM7, 1:400 dilution; catalog 103026), APC/Cyanine7 anti-CD279 (PD-1; 29F.1A12, 1:200 dilution; catalog 135224), PE/Cyanine7 anti-Tim3 (RMT3-23, 1:200 dilution; catalog 119716), PE anti-CD127 (Il7r; A7R34, 1:300 dilution; catalog 135009) (all from BioLegend), and Brilliant Violet 711 Annexin V (catalog 563972, BD Horizon, 1:300 dilution). LCMV GP33-specific CD8^^+^^ T cells were detected by tetramerization of GP33 monomers (supplied by the NIH Tetramer Core Facility) conjugated to streptavidin-APC (eBioscience).

Cells were then fixed and permeabilized for intracellular staining using the 10× Permeabilization Buffer (Invitrogen). Mouse intracellular antibodies used for staining throughout this study include APC anti–IFN-γ (XMG1.2, 1:200 dilution; catalog 505810), Brilliant Violet 650 anti-TNF (MP6-XT22, 1:400 dilution; catalog 506333), Brilliant Violet 785 anti-Tbet (4B10, 1:200 dilution; catalog 644835) PE/Dazzle 594 anti–Granzyme B (QA16A02, 1:200 dilution; catalog 372216), PE anti-Perforin (S16009A, 1:200 dilution; catalog 154306), Alexa Fluor 488 anti-Puromycin (2A4, 1:200; catalog 381506) (all from BioLegend), Alexa Fluor 488 anti–TCF-7/TCF-1 (S33-966, 1:200 dilution; catalog 567018, BD Biosciences), eFluor 660 anti-TOX (TXRX10, 1:200 dilution; catalog 50-6502-82, eBioscience), and Brilliant Ultra Violet 737 anti–Ki-67 (SolA15, 1:150 dilution; catalog 367-5698-82, eBioscience).

### *Cytotoxicity assay of P14 CD8^^+^^ T cells*.

For in vitro cytotoxicity assay, GP33-expressing CT2A glioma tumor cells (a gift from Hiroshi Nakashima, Harvard University, Boston, Massachusetts, USA) (52) were labeled with CFSE dye (1 μM, Sigma), then seeded into a 96-well, flat-bottom plate at a concentration of 60,000 cells per well in complete RPMI medium. The tumor cells were incubated at 37°C and 5% CO__2__ overnight to allow monolayer formation. The following day, each well was seeded with 60,000 P14 cells collected from either the acutely or chronically stimulated conditions at the indicated time point. The cocultured tumor cells and P14 cells were then incubated overnight (18–20 hours), followed by staining for spectral cytometry. Tumor cell killing activity was determined by measuring the number of dead tumor cells per 10 viable CD8^^+^^ T cells by spectral cytometry.

### *RNA-Seq*.

RNA was isolated from sorted P14 cell pellets using the Arcturus PicoPure RNA Isolation Kit (catalog KIT0204, Applied Biosystems). RNA samples were submitted for library prep and sequencing by Azenta Life Sciences. RNA-Seq data were analyzed by Partek Flow software. The generated FASTQ files were used for prealignment quality assurance/quality control, then aligned to the mouse mm10 genome by STAR. The resultant aligned reads were quantified to the mm10 RefSeq Transcripts using Quantify to annotation model, followed by filtering to exclude features where maximum < 50.0. DESeq2 analysis was performed first by normalization of the filtered gene counts for median ratio to compare among the P14 conditions. DEGs were determined as genes with fold change ≥ 2 and *P* < 0.05 between the compared groups. The DEGs were plotted into heatmaps and used for PCA compared with the transcriptional signatures of TEX subsets from acute or chronic LCMV infection in mice (28, 36) using Partek Flow software. GSEA for Hallmark and Gene Ontology pathways and the pan-cancer single-cell RNA-Seq gene signatures for human TILs (33) was performed using Partek Flow software. The lists of significant biomarker genes for TEX subsets were calculated using the normalized RNA counts of TEX subsets (generated in vivo) (28) on Partek (*P* < 0.05, fold change ≥ 2). Enrichment analyses using these transcriptional signatures of TEX subsets were performed on GSEA software (UCSD and Broad Institute, v4.3.3).

### *ATAC-Seq*.

P14 cells were isolated for ATAC-Seq using the Sony MA900 Cell Sorter, including Acute-7d or T__Dysf__ PD-1^^+^^Tim3^^+^^ on day 19 and resting T__Dysf__ cells isolated as PD-1^^+^^Tim3^^–^^ (Rest-SP) or PD-1^^+^^Tim3^^+^^ (Rest-DP) on day 23. ATAC was performed using Omni-ATAC, as previously described (53), with minor modifications. Briefly, nuclei were isolated from cells using ATAC lysis buffer (10 mM Tris-HCl pH 7.5, 10 mM NaCl, 3 mM MgCl__2__, 0.1% NP-40, 0.1% Tween 20, and 0.01% Digitonin) and then transposed for 30 minutes at 37°C using Tagment DNA TDE1 Enzyme (Illumina, 20034197). Transposed DNA was purified, and libraries were generated using Nextera XT DNA Library Preparation Kit (Illumina, FC-131-2001). The concentration and size distribution of libraries were determined by Qubit (Thermo Fisher Scientific) and Agilent Tapestation, respectively. The ATAC libraries were then sequenced by Azenta Life Sciences. The data were analyzed using cutadapt 1.18, Bowtie2, Picard 2.18.17, samtools, and deepTools 3.3.0. First, the FASTQ files were trimmed using cutadapt, then aligned to mouse mm10 genome assembly using Bowtie2. Next, duplicate reads were removed using Picard 2.18.17, and bigWig files were generated with deepTools. Differentially OCRs on day 19 were defined as regions with fold change ≥ 2 (for open in T__Dysf__) or ≤ –2 (for closed in T__Dysf__) and *P* < 0.01 for T__Dysf__ versus Acute-7d cells and plotted as heatmaps using Partek Flow software. PCA was performed using Partek to compare OCRs for Acute-7d, T__Dysf__, and Rest-SP or Rest-DP subsets to published chromatin landscapes for memory or exhausted CD8^^+^^ T cells (23) using the top 5,000 variable peaks. Accessible chromatin peaks were visualized using IGV software at the indicated genomic sites. To visualize chromatin accessibility transitions specific to T__Dysf__ PD-1^^+^^Tim3^^+^^ cells, we generated an alluvial plot. Peaks were classified as “open” or “closed” (stage 2) based on comparisons to the Acute-7d condition, using thresholds of adjusted *P* < 0.01 and fold change ≥ 2 or ≤ –2. These peaks were then evaluated in resting states (Rest-DP and Rest-SP) to assign stage 3 labels — open, closed, or unchanged — using the same statistical cutoffs. The Sankey-style plot depicts the number of peaks transitioning from the Acute-7d baseline (stage 1) through their T__Dysf__-specific state (stage 2) to their resting fate (stage 3), highlighting dynamic regulatory patterns across conditions. TF enrichment analysis was performed using HOMER (findMotifsGenome.pl) with the mm10 genome and the -size given option to preserve original region lengths.

### *Targeted DNA methylation sequencing*.

DNA was isolated from FACS-purified P14 cell pellets (sorted on day 19 of in vitro stimulation) and used for bisulfite conversion using the EZ DNA Methylation-Direct Kit (Zymo). PCR was then performed on the bisulfite-converted DNA at DMRs for key genes related to CD8^^+^^ T cell exhaustion states (*Tcf7*, *Ccr7*, *Pdcd1*). The following primers were used for each region: *mTcf7* (forward primer: 5′-GGTTAGTTTGAGTTTGGTTTAGAGTAGTGAG-3′, reverse primer: 5′-CCTCTTACCTAAATTTCCCTACAAAATACC-3′), *mCcr7* (forward primer 5′-GGAGTTTGGGATAAAAGTTTTTAATGG-3′, reverse primer: 5′-CCAAACCCACTCTAAACCCTATATTAC-3′), and *mPdcd1* (forward primer: 5′-GGTTGAGAGAGATTGAAATTAGGGTTAG-3′, reverse primer: 5′-CAACAAAACTAACAAACCTAAAACAACT-3′). Following PCR amplification, the amplicon size was verified by gel electrophoresis. Gel bands were excised, and amplicon DNA was purified from each region using the Zymoclean Gel DNA Recovery Kit (Zymo). The purified amplicon DNA was then used for library preparation using the Native Barcoding Kit 24 V14 (Oxford Nanopore Technologies) (9). The barcoded DNA library was loaded onto an R10 flow cell and run on the MinION Mk1B sequencer from Oxford Nanopore. FASTQ files generated from the sequencing run were used for downstream analysis for genome alignment and determining percentage of CpG methylation at each amplified region using a customized NanoEM pipeline (54).

### *Statistics*.

Statistical analyses were performed using GraphPad Prism v10. For flow cytometry and DNA methylation data analysis, comparisons between 2 groups were performed using the Mann-Whitney *U* test (unpaired, 2-sided). For comparison of 3 or more groups, 1-way ANOVA tests (for 1 factor) or 2-way ANOVA tests (for 2 factors) were performed with a Tukey’s multiple-comparison correction. Biological and technical replicates (*n*) are stated in the figures. All data were expressed as mean ± SEM, and a *P* value of less than 0.05 was considered statistically significant. Statistical significance for RNA-Seq data was determined by PCA, DESeq2 analysis, or GSEA using Partek Flow software (v12) or UCSD and Broad Institute GSEA software. Statistical significance for ATAC-Seq data was determined by PCA and DESeq2 analysis using Partek Flow software.

### *Study approval*.

All research conducted in this study complies with the ethical regulations under the approved protocols by The Ohio State University IACUC (IACUC #2019A00000055-R1) and the Institutional Biosafety Committee (IBC #2020R00000129).

### *Data availability*.

Values for all data points in graphs are reported in the Supporting Data Values XLS file. ATAC-Seq and RNA-Seq datasets have been deposited in the NCBI BioProject PRJNA1299473 and Gene Expression Omnibus under the accession codes GSE304670 and GSE304671, respectively.

## Author contributions

Conceptualization: HEG. Methodology: HEG, AY, AAS, and AL. Investigation: HEG, AY, AAS, AL, AMY, KP, AS, MRA, and EMO. Visualization: HEG, AY, AAS, and AL. Supervision: HEG. Writing — original draft: HEG, AY, AAS, and AL. Writing — review and editing: HEG, EMO, AY, AAS, and AL.

## Supplementary Material

Supplemental data

Supporting data values

## Version 1 10/08/2025

Electronic publication
