# Supplementary material for: Faithful modeling of terminal CD8+T cell dysfunction and epigenetic stabilization in vitro
Source: JCI Insight. 2025 Oct 8;10(19):e191220. doi: 10.1172/jci.insight.191220 (PMC12513483; doi:10.1172/jci.insight.191220)
Supplement: Supplemental data [file jciinsight-10-191220-s045.pdf]

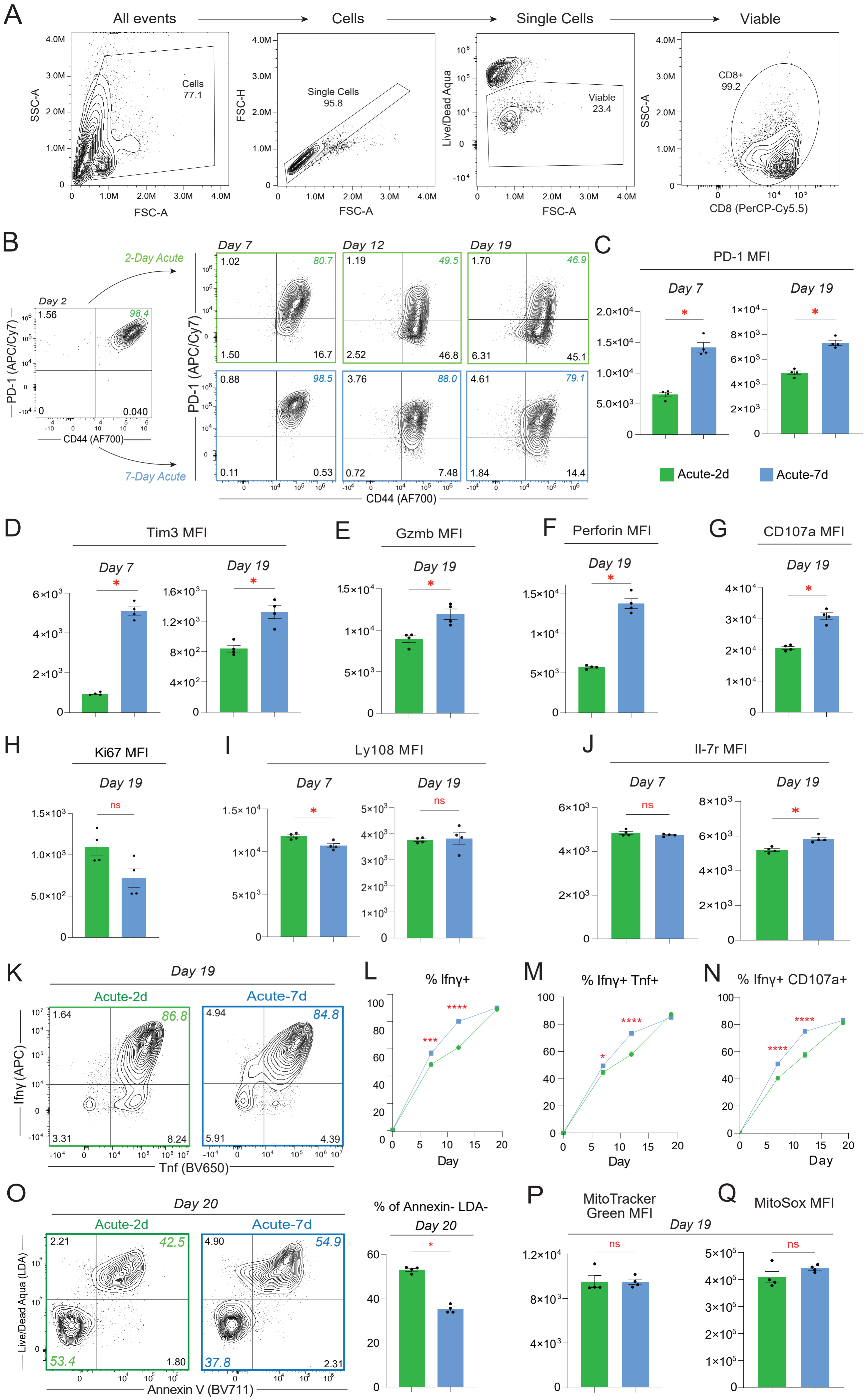

SUPPLEMENTAL FIGURE 1

Supplemental Fig. 1. (**A**) Gating strategy for *in vitro* stimulated P14 CD8+ T cells. (**B**) Representative FACs plots showing PD-1 and CD44 expression on Acute-2 and 7day P14 CD8+ T cells on days 2, 7, 12 and 19. (**C**) Expression level (gMFI) of PD-1and (**D**) Tim3 on days 7 and 19 on Acute-2d or -7d P14 cells. **(E)** Expression level (gMFI) of Gzmb; (**F**) Perforin; (**G**) CD107a and; (**H**) Ki67 after GP33 peptide rechallenge on day 19 on Acute-2d or -7d P14 cells. (**I**) Expression level (gMFI) of Ly108 and (**J**) Il7r on days 7 and 19 on Acute-2d or -7d P14 cells. (**K**) Representative FACS plots of Ifnγ and Tnf expression on P14 cells on day 19 after GP33 peptide rechallenge. (**L**) Longitudinal tracking of % total Ifnγ+; **(M) %** Ifnγ+ Tnf+; and **(N) %** Ifnγ+ CD107a+ P14 cells after GP33 peptide rechallenge at the indicated timepoints. **(O)** Representative FACs plots showing Live/Dead Aqua (LDA) and Annexin V staining on Acute-2 and 7day P14 CD8+ T cells on day 20 along with a summary bar graph. (**P**) Expression level (gMFI) of MitoTracker Green dye (for mitochondrial mass) and (**Q**) MitoSox dye (for mitochondrial reactive oxygen species, "ROS") within P14 cells on day 19. All $n = 4$ biological replicates, representative of two to three independent experiments. Adjusted P value ns>0.05, *$P<0.05$, **$P < 0.01$, ***$P < 0.001$, ****$P < 0.0001$. Comparisons were determined by two-way ANOVA (**L-N**), or the Mann–Whitney U test (unpaired, two sided) (**C-J, O-Q**). Error bars indicate mean ± SEM.

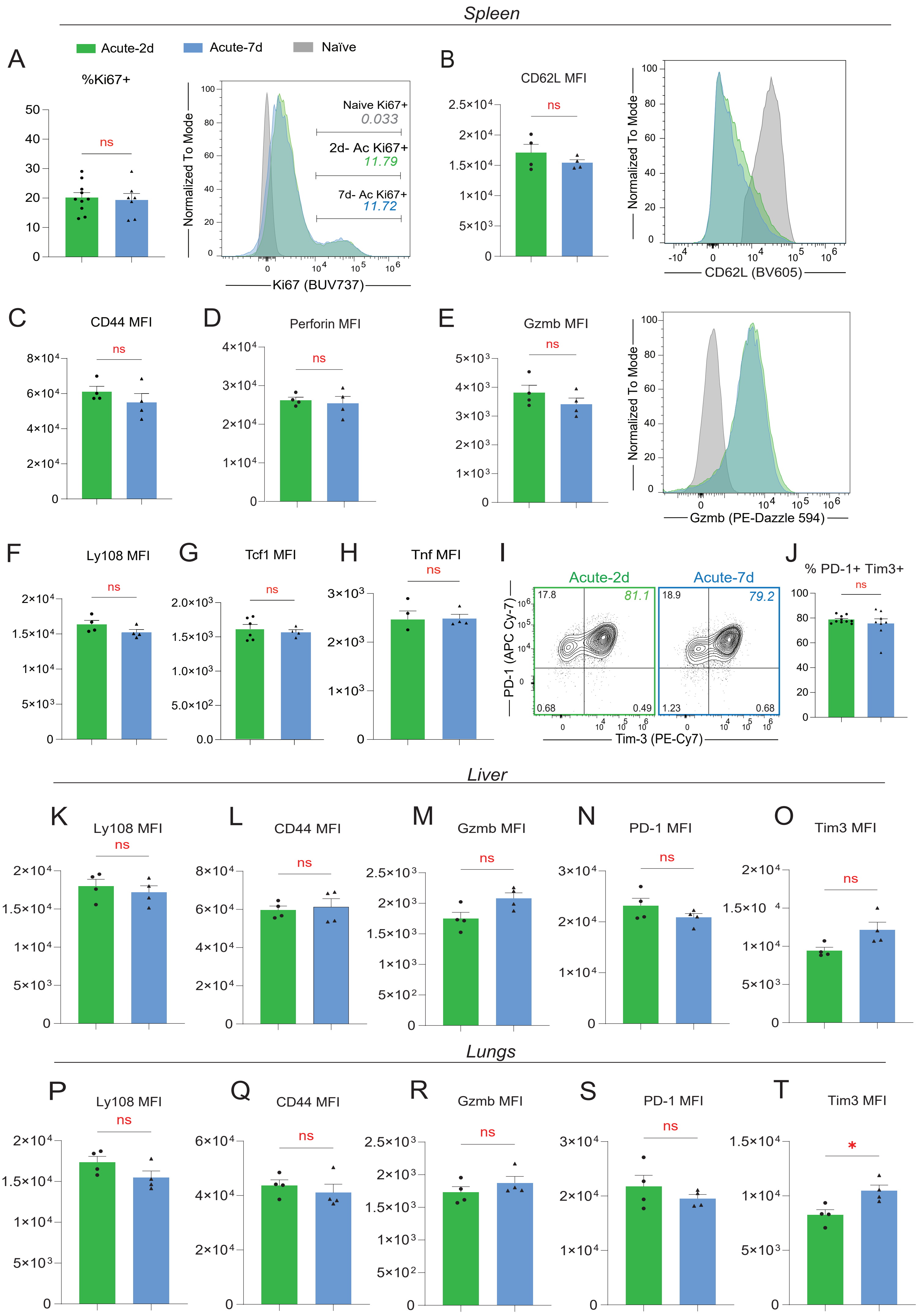

SUPPLEMENTAL FIGURE 2

Supplemental Fig. 2. (A) Bar graph showing the frequency of Ki67+ 2 or 7-day acute P14 CD8+ T cells from the spleens of C57BL/6 mice infected with acute LCMV (exact experiment design described in **Fig.1 G**) along with a representative histogram. **(B)** Bar graph and representative histogram showing the expression level (gMFI) of CD62L. **(C)** Bar graphs showing the expression levels (gMFI) of CD44, and **(D)** Perforin. **(E)** Bar graph and representative histogram showing the expression level (gMFI) of Gzmb. **(F)** Bar graphs showing the expression levels (gMFI) of Ly108; **(G)** Tcf1**;** and **(H)** Tnf. **(I)** Representative FACS plot and **(J)** summary bar graph of % PD-1+ Tim3+ P14 CD8+ T cells isolated from the spleens. **(K)** Bar graphs showing the expression levels (gMFI) of Ly108; **(L)** CD44; **(M)** Gzmb; **(N)** PD-1; and **(O)** Tim3 in P14 CD8+ T cells isolated from the livers. **(P)** Bar graphs showing the expression levels (gMFI) of Ly108; **(Q)** CD44; **(R)** Gzmb; **(S)** PD-1; and **(T)** Tim3 in P14 CD8+ T cells isolated from the lungs. For **A**, data were pooled from two independent experiments with $n = 3–4$ biological replicates per group for each experiment. For all other panels All $n = 4$ biological replicates, representative of two to three independent experiments. Adjusted P value ns>0.05 and $^{*}P < 0.05$. Comparisons were determined by the Mann-Whitney U test (unpaired, two sided). Error bars indicate mean ± SEM.

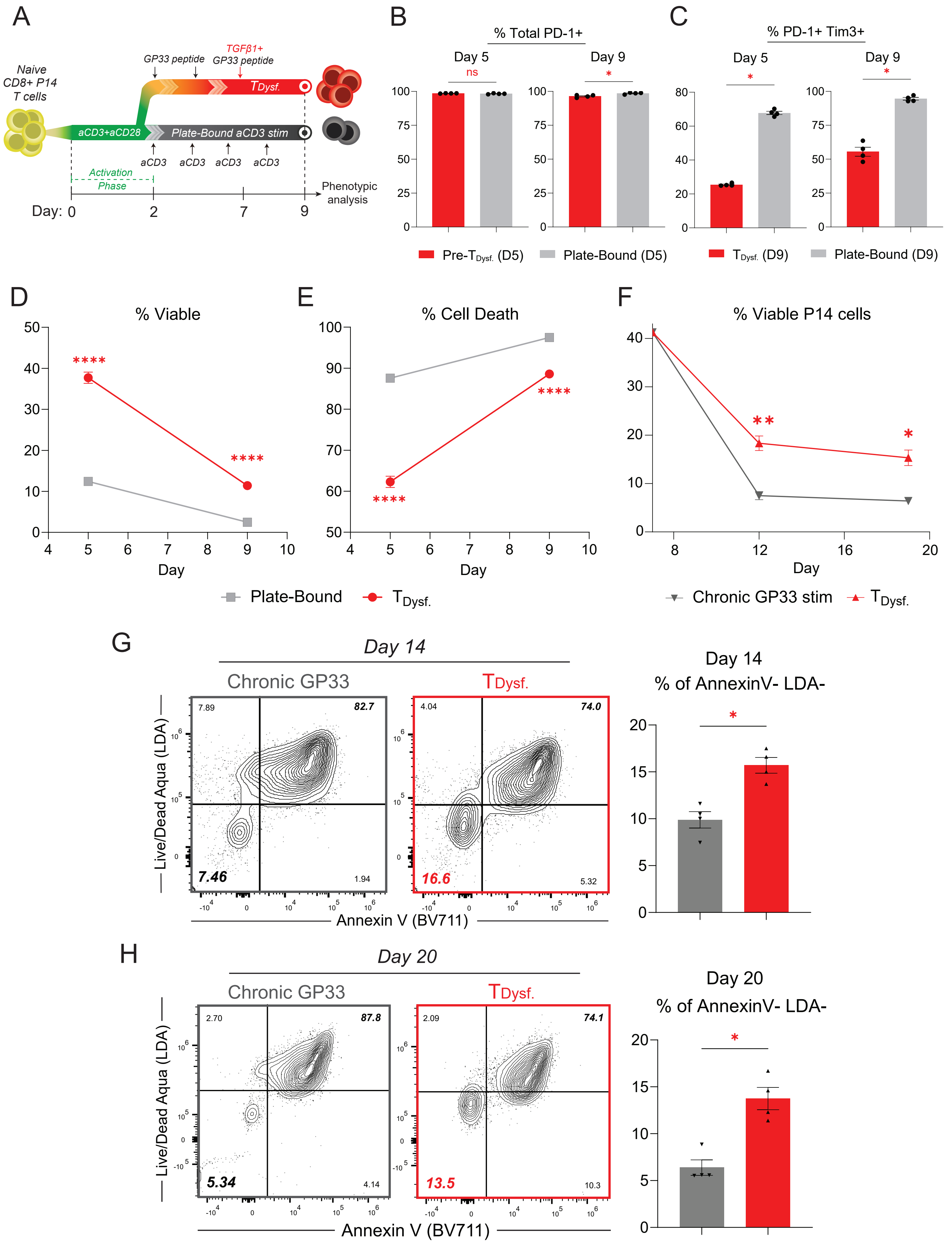

SUPPLEMENTAL FIGURE 3

Supplemental Fig. 3. (**A**) Schematic for *in vitro* stimulation of P14 cells comparing repeated plate-bound chronic ant-CD3 stimulation (d0-9) vs repeated GP33-stimulated from day 2-9 with chronic TGFβ1 exposure (red "T_{Dysf}"). Summary bar graph showing (**B**) frequency of Total PD-1 and (**C**) %PD-1+Tim3+ of T_{Dysf} vs plate-bound stimulated P14 CD8+ T cells on day 5 and day 9. Frequency of (**D**) viable, and (**E**) cell death of T_{Dysf} vs plate-bound stimulated P14 CD8+ T cells on day 5 and day 9 per 200 µl. (**F**) Longitudinal tracking of frequency of viable P14 cells per 200 µl from day 7-19 for Chronic GP33 stim (gray) or T_{Dysf} (red). Representative FACS plots and bar graphs of % of Annexin V- and live/dead (LDA-) expression on (**G**) Day 14, and (**H**) Day 20 for Chronic GP33 stim (gray) or T_{Dysf} (red). All $n = 4$ biological replicates, representative of two to three independent experiments. Adjusted P value $^{**}P < 0.01$, $^{***}P < 0.001$, $^{****}P < 0.0001$. Comparisons were determined by Two-way ANOVA (D-F), or the Mann–Whitney U test (unpaired, two sided) (**B-C, G-H**). Error bars indicate mean ± SEM.

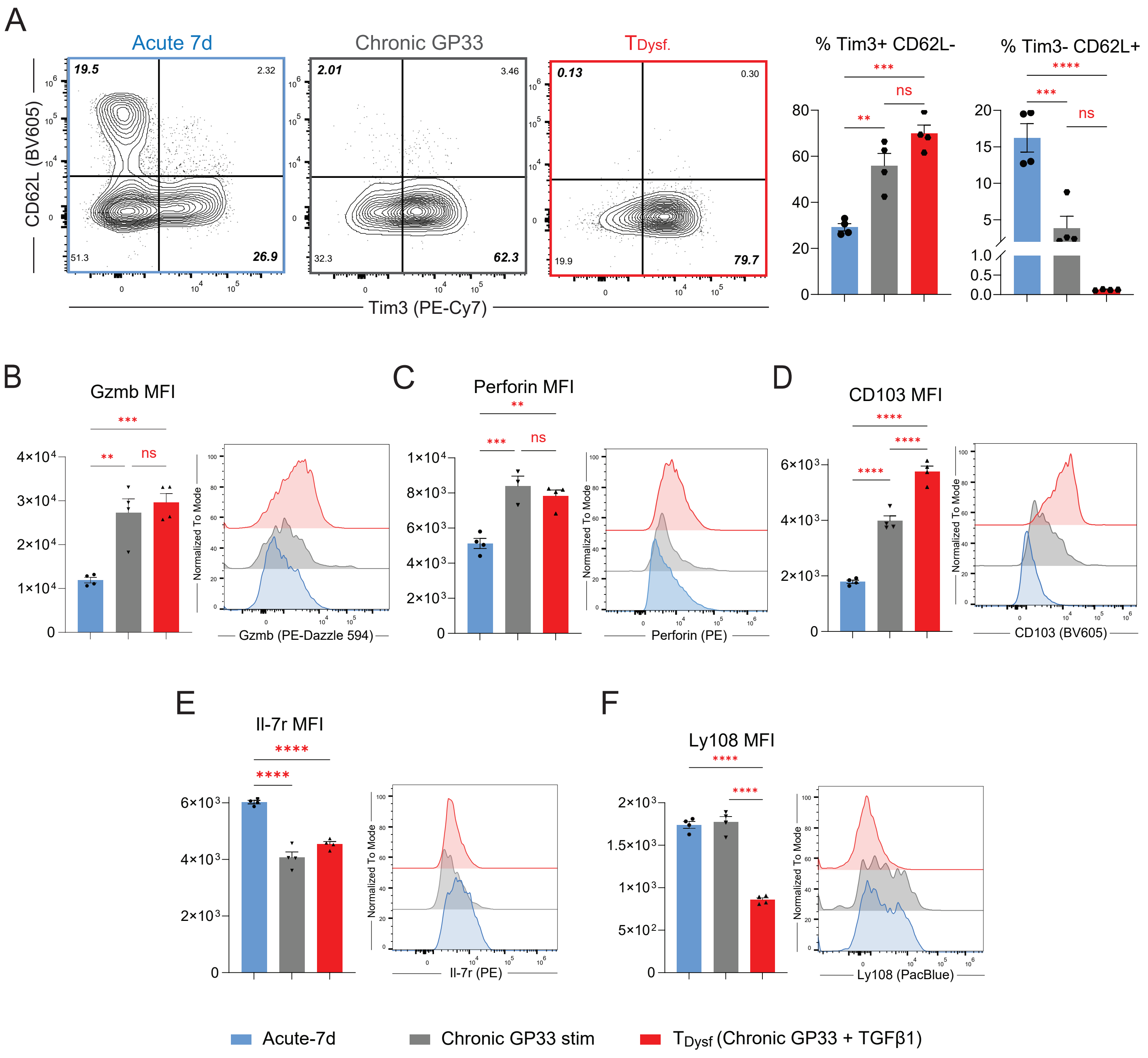

SUPPLEMENTAL FIGURE 4

Supplemental Fig. 4. (**A**) Representative FACS plots and summary bar graphs of Tim3 and CD62L expression (% Tim3+CD62L- and %Tim3-CD62L+) for Acute-7d (blue), Chronic GP33 stim (gray) or Chronic GP33+TGFβ1 (T_{Dysf} -red) on day 19. Expression level (gMFI) of (**B**) Gzmb, (**C**) Perforin, (**D**) CD103, (**E**) Il-7r, and (**F**) Ly108 within P14 cells on day 19 after GP33 peptide rechallenge. All $n = 4$ biological replicates, representative of two to three independent experiments. Adjusted P value $^{**}P < 0.01$, $^{***}P < 0.001$, $^{****}P < 0.0001$. Comparisons were determined by One-way ANOVA (**A-G**). Error bars indicate mean ± SEM.

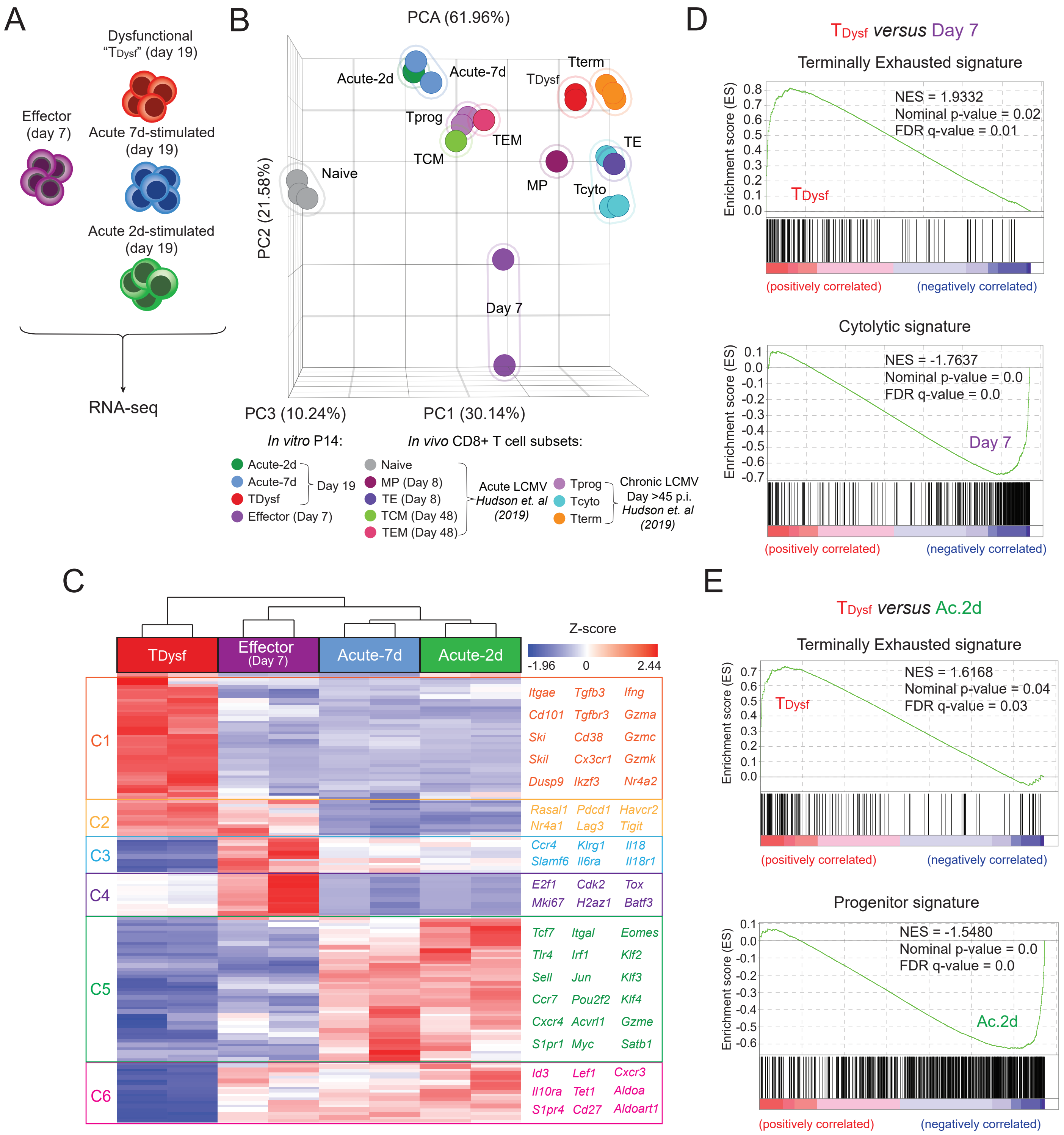

SUPPLEMENTAL FIGURE 5

Supplemental Fig. 5 (A) Schematic for RNA sequencing of *in vitro*-generated P14 cells that were FACS-purified on day 7 or day 19 of the model described in **Fig.1A** and **2A**. **(B)** Principal component analysis (PCA) plot comparing global transcriptional signatures of *in vitro*-generated P14 cells isolated on day 7 (Effector) or day 19 (Acute-2d, Acute-7d or dysfunctional "T_{Dysf}" cells) to published transcriptional signatures of exhausted PD-1+ CD8+ T cell subsets on day >45 of chronic LCMV infection (Progenitor "Tprog" CD101-Tim3-, Cytolytic "Tcyto" CD101-Tim3+, Terminally Exhausted "Tterm" CD101+Tim3+) from Hudson et. al (28), and naive, effector or memory CD8 T cell subsets on day 8 (Memory Precursor "MP" or Terminal Effector "TE") or day 48 post-acute LCMV infection (Central Memory "TCM", Effector Memory "TEM") from Hudson et. al (36). **(C)** Heatmap showing differentially expressed genes (DEGs) across the four P14 conditions and grouped into clusters based on shared expression patterns. **(D)** Gene set enrichment analysis (GSEA) plots for DEGs upregulated in T_{Dysf} versus Effector (Day 7), or (**E**) versus Acute-2d P14 cells in comparison to published signatures for TEX subsets from day >45 of chronic LCMV infection (28). *N=2* biological replicates per condition for RNA-seq. Statistical significance was determined by PCA (**B**) or DESeq2 analysis (**C**) using Partek software, or GSEA (**D-E**) using UC San Diego and Broad Institute GSEA software.

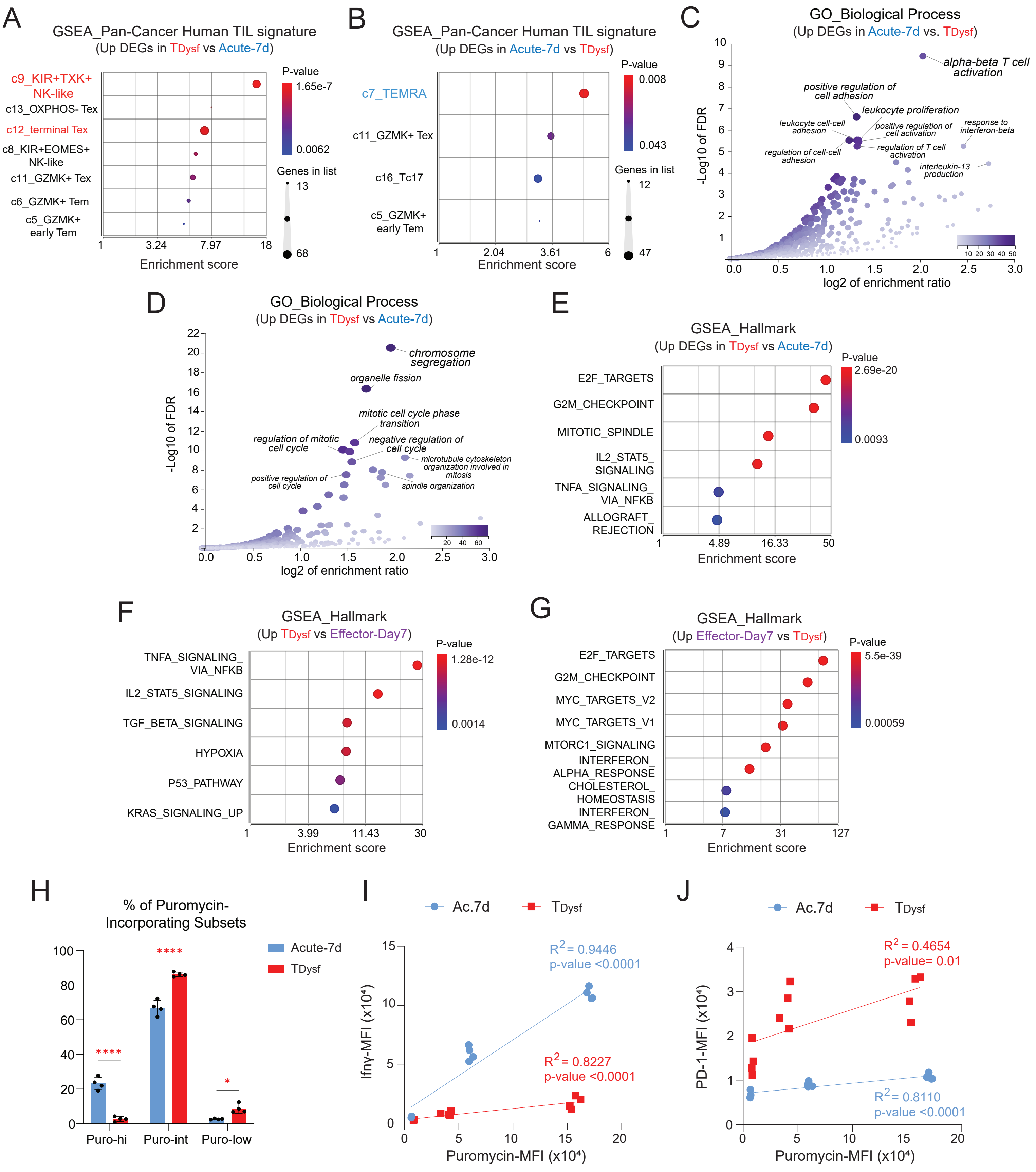

SUPPLEMENTAL FIGURE 6

Supplemental Fig. 6 (A) Gene set enrichment score plot for differentially expressed genes (DEGs) upregulated in T_{Dysf} versus Acute-7d, or **(B)** upregulated in Acute-7d versus T_{Dysf} compared to the pan-cancer human TILs gene signature (33). **(C)** Volcano plot for gene ontology (GO) enrichment of biological processes for DEGs upregulated in Acute-7d *versus* T_{Dysf}, or **(D)** upregulated in T_{Dysf} *versus* Acute-7d. **(E)** GSEA score plot compared to Hallmark gene signature for DEGs upregulated in T_{Dysf} versus Acute-7d, **(F)** upregulated in T_{Dysf} vs. Effector-Day 7, or **(G)** upregulated in Effector-Day 7 *versus* T_{Dysf}. **(H)** Bar graph showing frequencies of intracellular puromycin-incorporating subsets (high, intermediate and low) within P14 cells on day 19 after GP33 peptide rechallenge. **(I)** Summary plots showing intracellular levels of puromycin versus Ifnγ gMFI, or **(J)** PD-1 gMFI in P14 cells on day 19 after GP33 peptide rechallenge, with linear regression (R^2) and p-values listed. *N*=2 biological replicates for RNA-seq, or *n* = 4 biological replicates for **H-J**, representative of two to three independent experiments. Statistical significance for (**A-G**) was determined by GSEA or GO enrichment analysis using Partek software. Significance in (**H**) was determined by One-way ANOVA with Tukey's multiple comparisons, or (**I-J**) by simple linear regression. Adjusted P value $*P<0.05$, $****P < 0.0001$. Error bars indicate mean ± SEM.

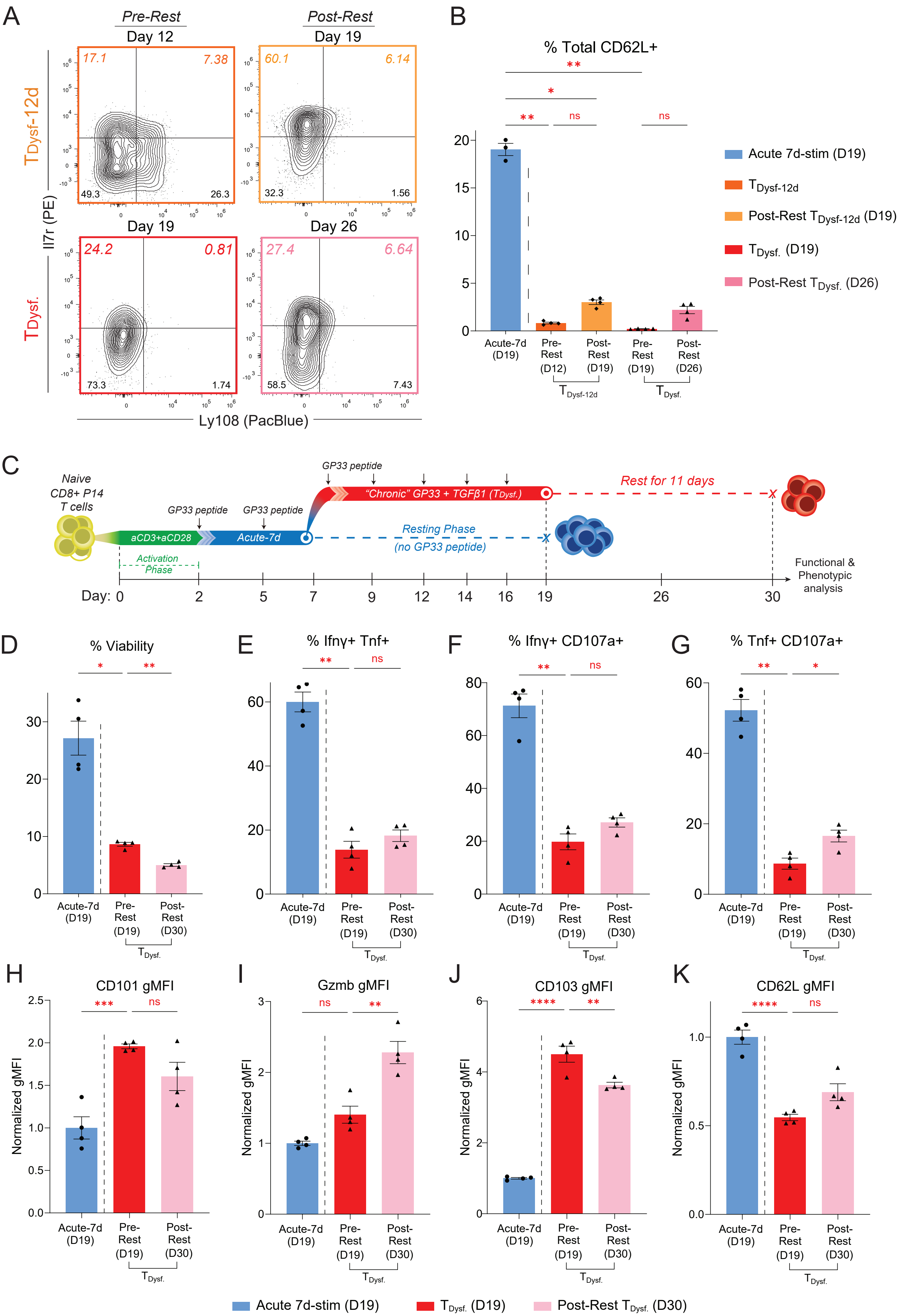

SUPPLEMENTAL FIGURE 7

Supplemental Fig. 7. (**A**) Representative FACS plots showing Il-7r and Ly108 expression for TDysf-12d pre-rest (day 12) and post-rest (day 19) vs TDysf. pre- and post-rest (on days 19 and 26, respectively). (**B**) Bar graph showing % of Total CD62L+ P14 cells. (**C**) Schematic showing resting phase of T_{Dysf} P14 cells under homeostatic conditions for 11 days (Day 19-30). Summary bar graph comparing the frequencies of (**D**) viability, (**E**) Ifnγ+Tnf, (**F**) Ifnγ+CD107a+, and (**G**) Tnf+CD107a+ P14 cells following GP33 peptide rechallenge for P14 CD8+ T cells on days 19 and 26. Expression level (normalized gMFI) of (**H**) CD101, (**I**) Gzmb, (**J**) CD103, (**K**) CD62L within P14 cells normalized to Acute-7d on day 19. All $n = 4$ biological replicates, representative of two to three independent experiments. Adjusted P value $^{**}P < 0.01$, $^{***}P < 0.001$, $^{****}P < 0.0001$. Comparisons were determined by One-way ANOVA (**A, C-L**). Error bars indicate mean ± SEM.

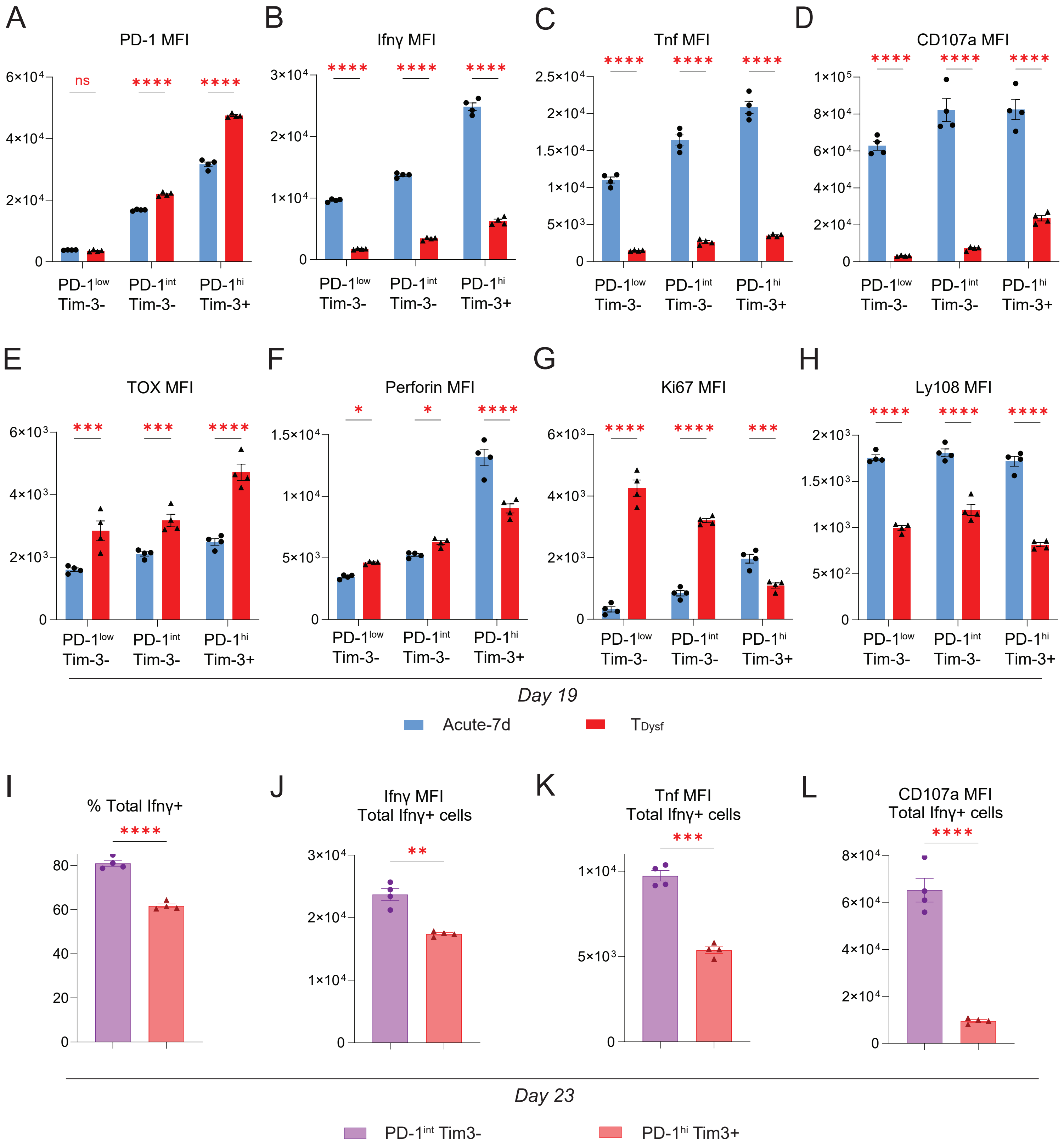

SUPPLEMENTAL FIGURE 8

Supplemental Fig. 8. (**A**) Bar graphs showing the expression level (gMFI) of PD-1, (**B**) Ifnγ, (**C**) Tnf, (**D**) CD107a, (**E**) TOX, (**F**) Perforin, (**G**) Ki67, and (**H**) Ly108 on P14 cells within the indicated PD-1/Tim3 subsets on day 19 after 5-hr GP33 peptide rechallenge. (**I**) Frequency of total Ifnγ+ P14 cells on day 23 after GP33 peptide rechallenge following the resting phase of T_{Dysf} cells under homeostatic conditions. (**J**) Expression level (gMFI) of Ifnγ, (**K**) Tnf, and (**L**) CD107a within the Ifnγ+ P14 cells on day 23 after GP33 peptide rechallenge of T_{Dysf} $PD1^{int}$ $Tim3^{-}$ and $PD1^{hi}$ $Tim3^{+}$ subsets. All *n* = 4 biological replicates, representative of two to three independent experiments. Adjusted P value **P<0.05*, ***P < 0.01*, ****P < 0.001*, *****P < 0.0001*. Comparisons were determined by One-way ANOVA (**A-H**) or Mann-Whitney U test (unpaired, two sided) (**I-L**). Error bars indicate mean ± SEM.

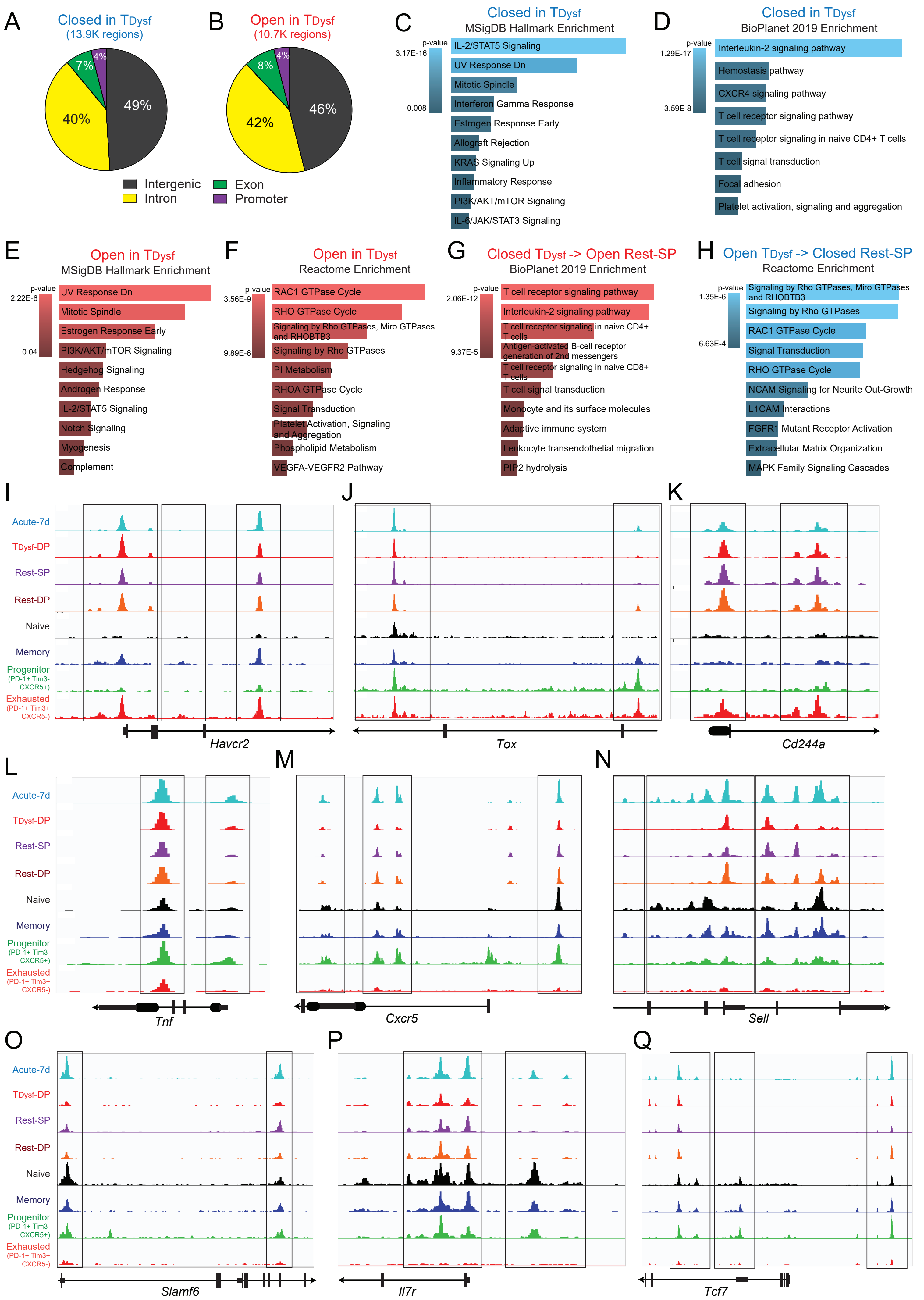

SUPPLEMENTAL FIGURE 9

Supplemental Fig. 9 (**A**) Pie chart showing frequency distribution of genomic sites within Closed chromatin regions in T_{Dysf}-DP (PD-1+Tim3+) P14 CD8 T cells, or (**B**) Open regions in T_{Dysf}-DP cells on Day 19. (**C**) Pathway enrichment analysis of genes within Closed regions in T_{Dysf}-DP cells compared to the MSigDB Hallmark gene signature, or (**D**) BioPlanet 2019 signature. (**E**) Pathway enrichment analysis of genes within Open regions in T_{Dysf}-DP cells on Day 19 vs Acute-7d cells compared to the MSigDB Hallmark gene signature, or (**F**) Reactome signature. (**G**) Pathway enrichment analysis for genes within Closed regions in TDysf-DP cells that became Open in Resting SP (PD-1+Tim3-) cells on Day 23 compared to the BioPlanet 2019 signature, or (**H**) Open regions in TDysf-DP cells that became Closed in Resting SP cells compared to the Reactome signature. (**I**) Representative IGV snapshots of mapped accessible chromatin peaks within *in vitro* P14 or *in vivo* naive, memory or exhausted subsets (44) at gene loci for *Havcr2*, (**J**) *Tox*, (**K**) *Cd244a*, (**L**) *Tnf*, (**M**) *Cxcr5*, (**N**) *Sell*, (**O**) *Slamf6*, (**P**) *Il7r*, and (**Q**) *Tcf7*. *N*=2 biological replicates for ATAC-seq. Statistical significance for (**C-H**) was determined by Enrichr, with adjusted p-value of enriched pathways <0.05.

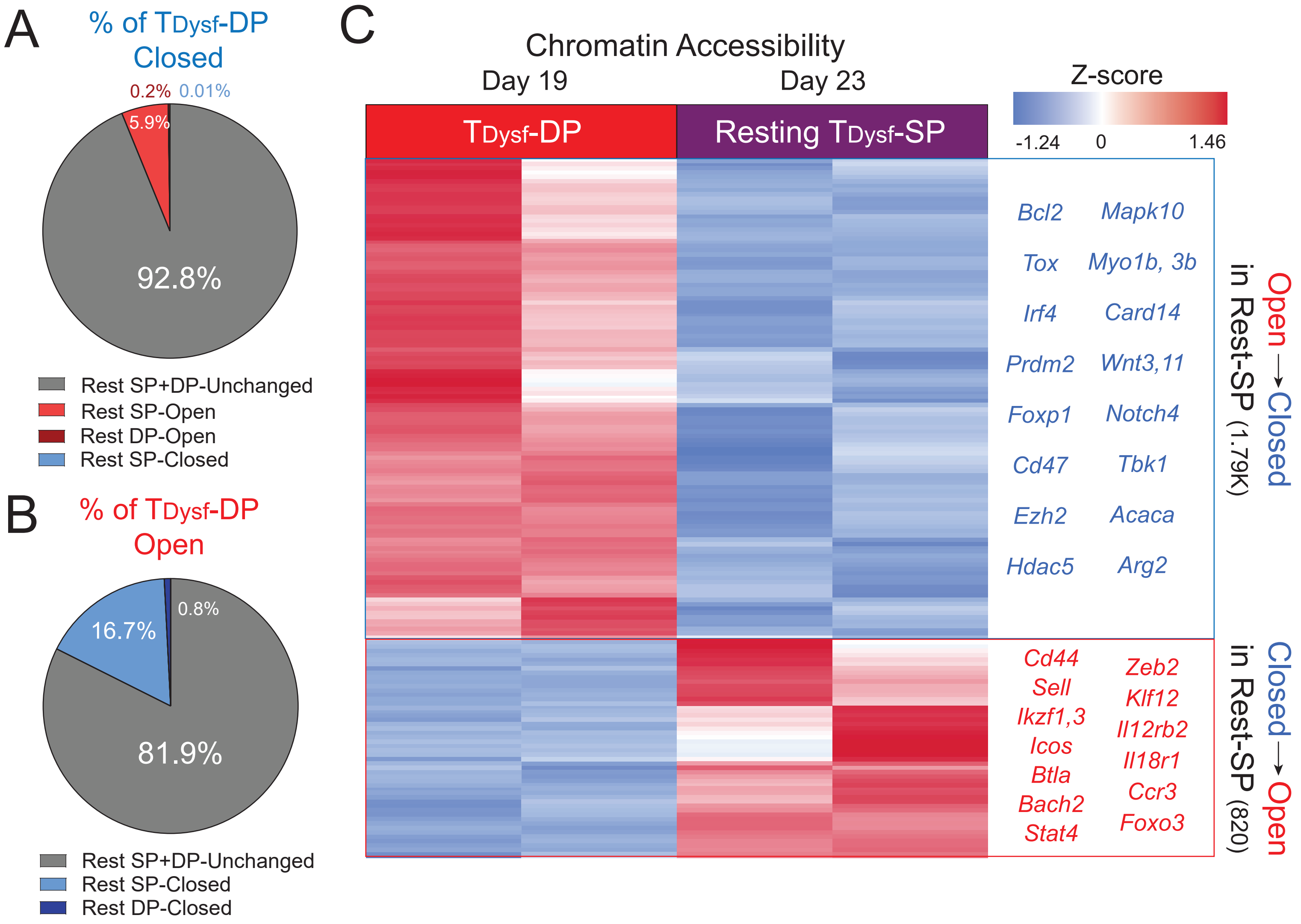

SUPPLEMENTAL FIGURE 10

Supplemental Fig. 10 (A) Pie chart showing frequency of differentially open chromatin regions (OCRs) that were Closed in T_{Dysf}-DP cells compared to Acute-7d cells on Day 19, which remained Unchanged or became more Open or Closed in Resting T_{Dysf} cells on Day 23, and **(B)** Open OCRs in T_{Dysf}-DP Day 19 cells that remain Unchanged or became more Closed in Resting T_{Dysf} cells. **(C)** Heatmap showing differentially OCRs between T_{Dysf}-DP cells on Day 19 and Resting T_{Dysf}-SP cells on Day 23 with example genes listed. Statistical significance for **(C)** was determined by DESeq2 analysis using Partek software.

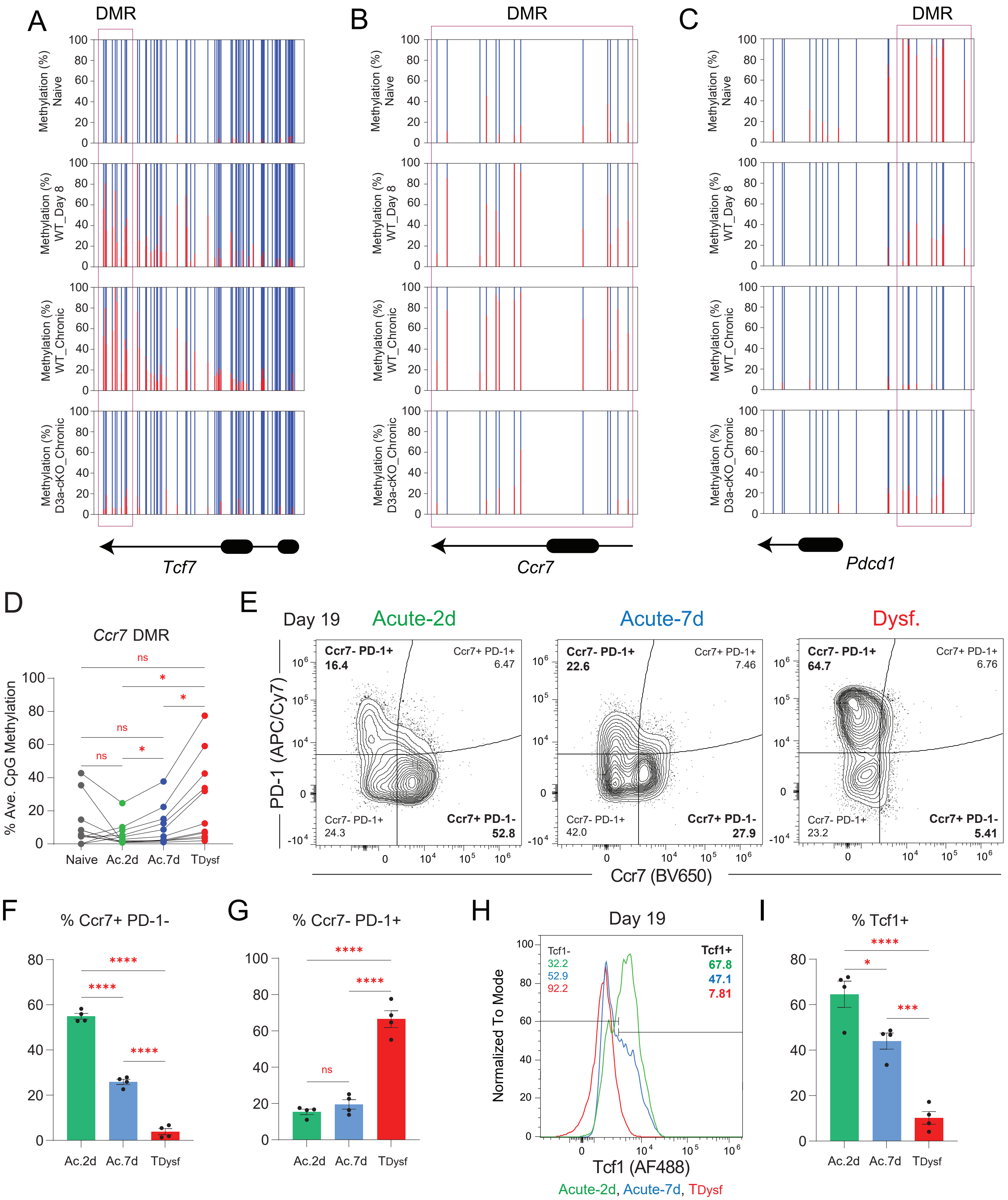

SUPPLEMENTAL FIGURE 11

Supplemental Fig. 11 (A) CpG methylation plots showing differentially methylated regions (DMRs) at gene loci for *Tcf7*, (**B**) *Ccr7*, and (**C**) *Pdcd1* within LCMV-specific CD8+ T cells at effector (Day 8) and chronic (Day 35) stages of LCMV infection from wild-type (WT) or Dnmt3a-conditional knockout (D3a-cKO) mice from Ghoneim et. al (8). Vertical lines represent individual CpG sites in the loci, while the ratio of blue-to-red indicates % unmethylated *versus* methylated reads, respectively. (**D**) % methylation of individual CpG sites at the DMR at *Ccr7* locus within naive or *in vitro*-generated P14 cells on day 19 ("Acute-2d", "Acute-7d", or "T_{Dysf}"). (**E**) Representative FACS plots showing expression of PD-1 and Ccr7, and (**F**) bar graph showing frequency of Ccr7+ PD-1-, or (**G**) Ccr7- PD-1+ P14 cells on day 19. (**H**) Representative histogram and (**I**) bar graph showing frequency of Tcf1+ P14 cells on day 19. *N*=2 biological replicates for DNA methylation sequencing, or *n*=4 biological replicates for (**E-I**), representative of two to three independent experiments. Comparisons in (**D**) were determined by repeated-measures one-way ANOVA, or (**F-G, I**) ordinary one-way ANOVA analysis with Tukey's multiple comparisons test. Adjusted P value **P<0.05*, ***P<0.01*, ****P < 0.001*, *****P < 0.0001*. Error bars indicate mean ± SEM.

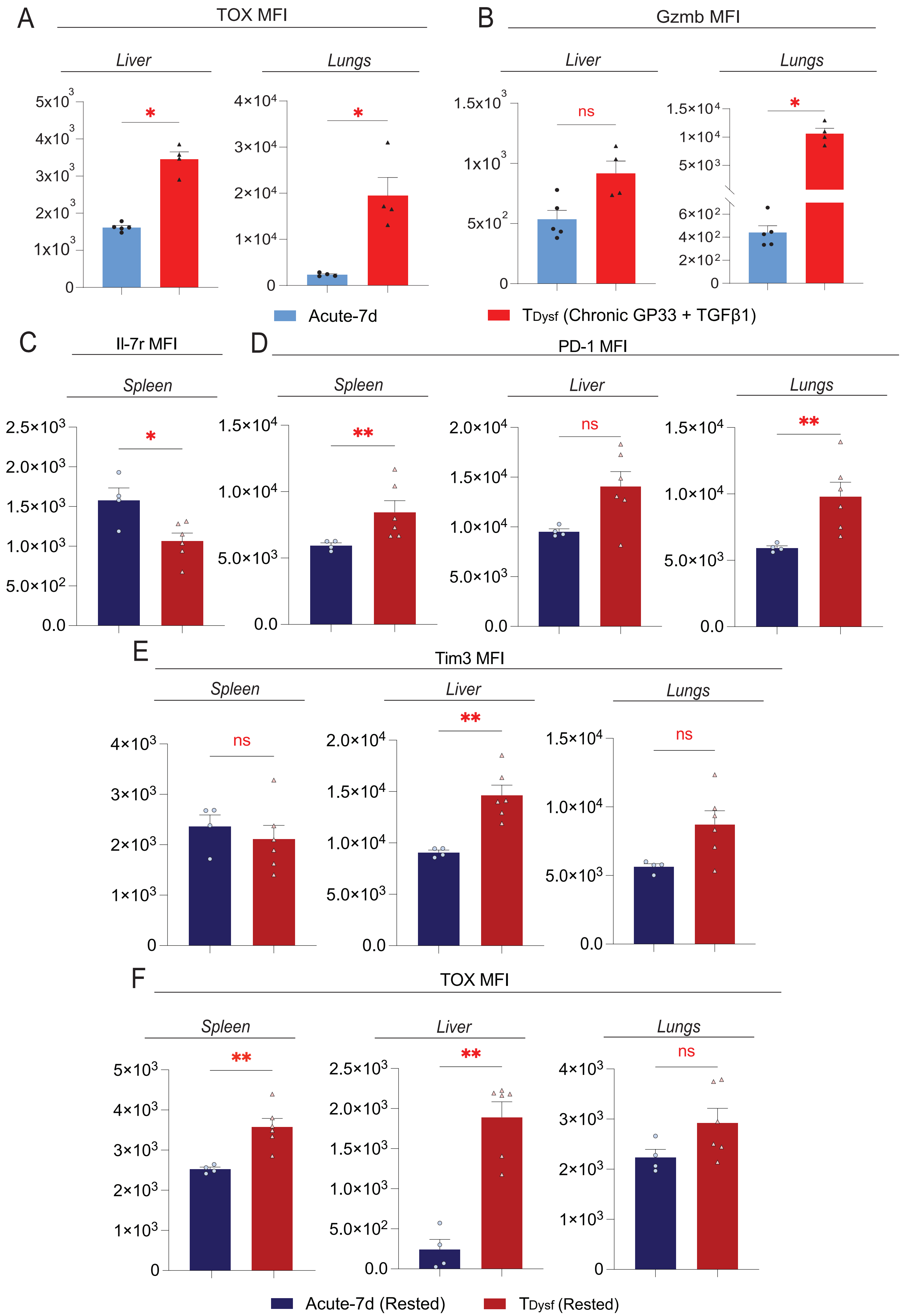

SUPPLEMENTAL FIGURE 12

Supplemental Fig. 12 (**A**) Bar graph showing the expression level (gMFI) of TOX and (**B**) Gzmb in P14 cells isolated from the livers and lungs of LCMV Armstrong infected mice (detailed explanation of experimental design for panels **A** and **B** in **Fig.7A**). (**C**) Bar graph showing the expression level (gMFI) of Il-7r in P14 cells isolated from the spleens of LCMV Armstrong infected mice (detailed explanation of experimental design for panels **C-F** in **Fig.7H**). (**D**) Bar graph showing the expression level (gMFI) of PD-1; (**E**) Tim-3; and (**F**) TOX in P14 cells isolated from the spleens, livers, and lungs. $N = 4\text{-}6$ biological replicates, representative of two to three independent experiments. Adjusted P value **P<0.05 and* ***P <0.01*. Comparisons were determined by the Mann-Whitney U test (unpaired, two sided). Error bars indicate mean ± SEM.
